# Interfacial Tensions, Solubilities, and Transport Properties of the H_2_/H_2_O/NaCl System: A Molecular Simulation Study

**DOI:** 10.1021/acs.jced.2c00707

**Published:** 2023-01-11

**Authors:** W. A. van Rooijen, P. Habibi, K. Xu, P. Dey, T. J. H. Vlugt, H. Hajibeygi, O. A. Moultos

**Affiliations:** † Reservoir Engineering, Geoscience and Engineering Department, Faculty of Civil Engineering and Geosciences, Delft University of Technology, Stevinweg 1, 2628CN, Delft, The Netherlands; ‡ Engineering Thermodynamics, Process and Energy Department, Faculty of Mechanical, Maritime and Materials Engineering, Delft University of Technology, Leeghwaterstraat 39, 2628CB, Delft, The Netherlands; ¶ Department of Materials Science and Engineering, Faculty of Mechanical, Maritime and Materials Engineering, Delft University of Technology, Mekelweg 2, 2628CD, Delft, The Netherlands

## Abstract

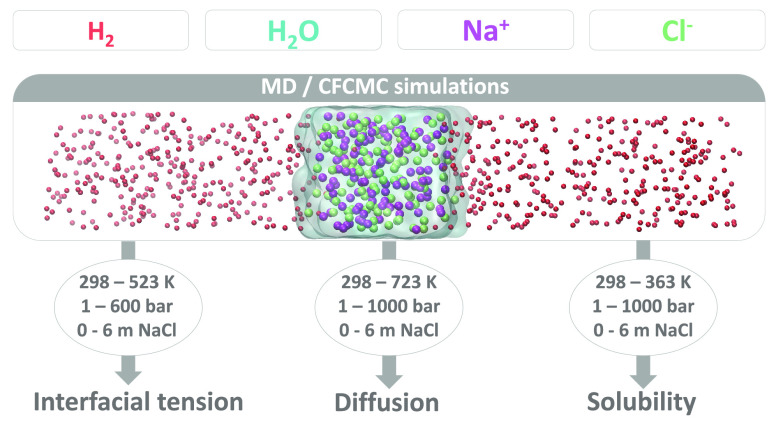

Data for several key thermodynamic and transport properties needed for technologies using hydrogen (H_2_), such as underground H_2_ storage and H_2_O electrolysis are scarce or completely missing. Force field-based Molecular Dynamics (MD) and Continuous Fractional Component Monte Carlo (CFCMC) simulations are carried out in this work to cover this gap. Extensive new data sets are provided for (a) interfacial tensions of H_2_ gas in contact with aqueous NaCl solutions for temperatures of (298 to 523) K, pressures of (1 to 600) bar, and molalities of (0 to 6) mol NaCl/kg H_2_O, (b) self-diffusivities of infinitely diluted H_2_ in aqueous NaCl solutions for temperatures of (298 to 723) K, pressures of (1 to 1000) bar, and molalities of (0 to 6) mol NaCl/kg H_2_O, and (c) solubilities of H_2_ in aqueous NaCl solutions for temperatures of (298 to 363) K, pressures of (1 to 1000) bar, and molalities of (0 to 6) mol NaCl/kg H_2_O. The force fields used are the TIP4P/2005 for H_2_O, the Madrid-2019 and the Madrid-Transport for NaCl, and the Vrabec and Marx for H_2_. Excellent agreement between the simulation results and available experimental data is found with average deviations lower than 10%.

## 1 Introduction

Due to the vastly growing global energy demand and the resulting climate change, a transition from fossil-fuel based energy production to clean renewable energy production is crucial.^1,2^ As a green energy carrier, hydrogen (H_2_) plays a crucial role in this transition because of its high gravimetric energy density and clean combustion products.^3−5^ Important technologies in the H_2_ value chain include underground H_2_ storage^6−9^ and H_2_O electrolysis.^10,11^ To enable the design and optimization of these technologies, accurate knowledge of thermodynamic, interfacial, and transport properties of H_2_ is essential.^9,12−15^ More specifically, the diffusivities and solubilities of H_2_ in aqueous solutions, and the interfacial tensions of H_2_ gas in contact with aqueous electrolyte solutions are crucial properties. The interplay of these properties determines the efficiency of the technologies, and allows for accurate predictions of the processes involved, which are, e.g., required for safety. These properties depend on pressure, temperature, and salt concentration.^16^ H_2_ technologies cover a wide range of operational conditions. For example, in underground H_2_ storage sites, the pressure, temperature, and salt molality can be as high as 300 bar, 333 K, and 5 mol NaCl/kg H_2_O, respectively.^7^ Typically, H_2_O electrolyzers operate at atmospheric pressure, temperatures of ca. (348 to 372) K, and molalities of ca. (3 to 4) electrolyte/kg H_2_O.^17,18^ Other types of electrolysis require much higher pressures and temperatures, i.e., up to 700 bar and 1400 K, respectively.^19−21^ Thus, to cover the conditions for important H_2_ applications, the interfacial tensions, self-diffusivities, and solubilities need to be available for a very wide range of pressures, temperatures, and salt concentrations.

Traditionally, these thermophysical properties are measured experimentally.^22−24^ Nevertheless, only a small number of experimental studies on the interfacial tension of H_2_/pure H_2_O^22,25−28^ is available, while only two studies report measurements of interfacial tension of H_2_/aqueous solutions (with NaCl and NaCl+KCl).^27,28^ These experiments are performed by using the capillary rise^29^ and the pendant drop^25,27,28,30^ techniques. Interfacial tensions of H_2_/aqueous solutions are reported for temperatures up to 423 K, pressures up to 345 bar, and molalities of up to 5 mol (NaCl + KCl)/kg H_2_O. As far as the solubility of H_2_ in aqueous NaCl solutions is concerned, for an overview of the available experimental data the reader is referred to the works of Chabab et al.,^31^ Torín-Ollarves and Trusler,^32^ and Ansari et al.^33^ Although a lot of experimental data are available for H_2_ in pure H_2_O, solubility measurements of H_2_ in aqueous NaCl solutions are scarce, and in many cases conflicting.^23,31,34−38^ The two main sources of experimental data of solubilities of H_2_ in aqueous solutions at concentrations above 1 mol NaCl/kg H_2_O, at temperatures above 300 K, and at pressures above 10 bar by Torín-Ollarves and Trusler,^32^ and Chabab et al.^31^ show conflicting results as the measured solubilities differ by ca. 30%. For the self-diffusivity of H_2_ in H_2_O, experimental data are available^24,39−46^ but mostly at atmospheric pressure and for limited temperatures below 340 K. Similarly to the solubilities, the experimental measurements also differ by up to 70%. To the best of our knowledge, no experimental data are available for the self-diffusivity of H_2_ in aqueous NaCl solutions.

Based on the available experimental data, it is evident that only a limited range of the required interfacial tensions, solubilities, and self-diffusivities of the H_2_/H_2_O/NaCl system has been measured, while in some cases, there are significant discrepancies between the data reported from different sources. The reason for the scarcity of and deviation in the data may be that experimental measurements are rather challenging and expensive to perform, especially at high pressures and temperatures. To this end, a widely used complementary approach for obtaining thermophysical data is molecular simulation, especially at conditions which are challenging for experimental measurements.

Numerous studies have used Molecular Dynamics (MD) to compute the interfacial tension of gases (e.g., CO_2_, CH_4_) and liquids (e.g., hydrocarbons) in contact with H_2_O (pure or saline).^47−57^ However, no molecular simulation studies on the interfacial tension of H_2_ and aqueous solutions are available. MD simulations have also been performed to compute self-diffusivities (i.e., self-diffusion coefficients) of H_2_ in pure H_2_O.^58−62^ Recently, Tsimpanogiannis et al.^60^ reported such data for pressures in the range of (1 to 2000) bar and temperatures in the range of (275 to 975) K spanning vapor, liquid, and supercritical H_2_O. The Marx,^63^ Vrabec,^64^ Buch,^65^ Hirschenfelder,^66^ Cracknell,^67^ and Silvera-Goldman^68^ H_2_ force fields were used in combination with the TIP4P/2005^69^ H_2_O force field. The Buch^65^ and Vrabec^64^ H_2_ force fields were shown to yield the best agreement with experimental data. In contrast, self-diffusivities of H_2_ in aqueous NaCl solutions computed from MD simulations have not yet been reported, while there are a few studies available reporting computations of self-diffusivities of CO_2_ in aqueous NaCl solutions.^70−73^ Lopez-Lazaro et al.^74^ have computed solubilities of H_2_ in aqueous NaCl solutions using Monte Carlo (MC) simulations for molalities up to a maximum of 2 mol NaCl/kg H_2_O. To the best of the authors’ knowledge, this was the only molecular simulation study on H_2_ solubilities in aqueous NaCl solutions. Molecular simulations have been used for computing solubilities of other gases (e.g., CO_2_, CH_4_) in water and aqueous NaCl solutions.^52,57,75−77^

Despite the urgency and importance of reliable data of interfacial tension of H_2_ in contact with aqueous NaCl solutions, self-diffusivity of H_2_ in aqueous NaCl solutions, and solubility of H_2_ in aqueous NaCl solutions, only very limited experimental and simulation studies are available. The objective of this work is to generate reliable data for these properties for a wide range of conditions relevant to H_2_ technologies, such as underground H_2_ storage and H_2_O electrolysis. We present new data sets of (a) interfacial tensions of H_2_ and aqueous NaCl solutions for temperatures, pressures, and molalities of (298 to 523) K, (1 to 600) bar, and (0 to 6) mol NaCl/kg H_2_O, respectively, (b) self-diffusivities of H_2_ in aqueous NaCl solutions for temperatures, pressures, and molalities of (298 to 723) K, (1 to 1000) bar, and (0 to 6) mol NaCl/kg H_2_O, respectively, and (c) solubilities of H_2_ in aqueous NaCl solutions for temperatures, pressures, and molalities of (298 to 363) K, (1 to 1000) bar and (0 to 6) mol NaCl/kg H_2_O, respectively. The interfacial tensions and self-diffusivities are computed using MD simulations, and the solubilities are computed using CFCMC^78−80^ simulations. Densities and viscosities of the aqueous NaCl solutions are also computed for a wide range of conditions and are compared to available experimental data. The TIP4P/2005^69^ force field is used for H_2_O, the Madrid-2019^81^ force field for NaCl, and the Vrabec^64^ and Marx^63^ force fields are used for H_2_. A modified version of the Madrid-2019 force field by Vega and co-workers^82^ (i.e., the Madrid-Transport^77,82^), optimized for viscosities of aqueous NaCl solutions for salinities up to the experimental solubility limit, is also used.

The paper is structured as follows. Details of the force fields used and the molecular simulation techniques are given in section 2. In section 3, the computed interfacial tensions, viscosities, densities, self-diffusivities, and solubilities obtained are presented and compared with experimental data when possible. Finally, concluding remarks are presented in section 4. All data computed in this study are provided in a tabulated format as Supporting Information.

## 2 Methodology

### 2.1 Force Fields

The four-site TIP4P/2005^69^ force field is used to model H_2_O. Previous studies have shown that this force field can accurately capture thermodynamic, transport, and interfacial properties of pure H_2_O and H_2_O/NaCl solutions in contact with gases for a wide range of conditions.^47,49,60,83−88^ For the Na^+^ and Cl^–^ ions, the Madrid-2019^81^ force field is used, which is parametrized for the TIP4P/2005 H_2_O model.^89^ A new version of the Madrid-2019 force field (i.e., Madrid-Transport force field^77,82^) is currently being developed by Vega and co-workers,^82^ which performs better for transport properties, especially at high NaCl molalities. The difference of Madrid-Transport from Madrid-2019 is that ion charges are scaled by 0.75 instead of 0.85, and the Lennard-Jones (LJ) parameters are slightly altered. Interfacial tensions and self-diffusivities are computed using the single-site Vrabec^64^ H_2_ force field, while for the solubilities of H_2_ in the aqueous NaCl solutions the three-site Marx^63^ model is used. Tsimpanogiannis et al.^84^ showed that the Vrabec^64^ H_2_ force field yields very accurate self-diffusivities of H_2_ in pure TIP4P/2005 H_2_O. The solubilities computed using the Vrabec^64^ force field deviate from experimental data of H_2_ in pure water by ca. 50%. In sharp contrast, the solubilities computed using the Marx^63^ force field show excellent agreement with experimental data. A comparison of the solubilities computed using the Marx and Vrabec force fields in pure TIP4P/2005 H_2_O are listed in Table S1 and shown in Figure S1 of the Supporting Information. At low temperatures, H_2_ exhibits quantum effects which can be accounted for by using potentials such as the Feynman-Hibbs effective interaction potential.^90−92^ At the temperatures considered in this work (i.e., 298 K and above) these quantum effects can be neglected.^93^ All force field parameters are listed in Tables S2–S5 in the Supporting Information. A list of the chemical formulas, CAS numbers, and force fields of all species studied here is shown in Table 1.

**Table 1:** Details of All the Species Simulated in This Work

Component	Chemical formula	CAS number	Force field
Water	H_2_O	7732-18-5	TIP4P/2005^69^
Hydrogen	H_2_	133-74-0	Vrabec^64^
Hydrogen	H_2_	133-74-0	Marx^63^
Sodium	Na^+^	7440-23-5	Madrid-2019^81^
Sodium	Na^+^	7440-23-5	Madrid-Transport^77,82^
Chloride	Cl^–^	7782-50-5	Madrid-2019^81^
Chloride	Cl^–^	7782-50-5	Madrid-Transport^77,82^

### 2.2 Molecular Simulation Details

#### 2.2.1 MD Simulations

The MD simulations are used to calculate (a) the interfacial tensions of H_2_ in contact with aqueous NaCl solutions, (b) self-diffusivities of H_2_ in aqueous NaCl solutions, (c) densities, and (d) viscosities of aqueous NaCl solutions. For all MD simulations, the Large-scale Atomic/Molecular Massively Parallel Simulator (LAMMPS)^94^ is used (version 29 Sep 2021). For the integration of the equations of motion, the velocity-Verlet algorithm is used with a time step of 1 fs. The bond length and bending angle of H_2_O are fixed using the SHAKE algorithm.^94,95^ The intermolecular interactions are described by Lennard-Jones and Coulombic interaction potentials. The Lorentz–Berthelot combining rules^96^ are used for interactions between different types of molecules, with the exception of Na^+^–H_2_O, Na^+^–Cl^–^, and Cl^–^–H_2_O LJ interactions as specified in Table S5. Long-range electrostatic energies are computed using the particle–particle particle–mesh (PPPM) method^97,98^ with a relative error^99^ of 10^–5^. The temperature and pressure are regulated by the Nosé–Hoover thermostat and barostat.^97^ Initial configurations are created using the PACKMOL software.^100^ Periodic boundary conditions are imposed in all directions. All MD simulations for a specific set of conditions are repeated 5 times using different initial velocity distributions from which the average quantities are calculated. The reported uncertainties are standard deviations from the results of these 5 simulations.

#### 2.2.2 Computation of Interfacial Tension

The following procedure is used for computing the interfacial tensions: An initial configuration is created by combining separately equilibrated bulk phases of aqueous sodium chloride solutions and H_2_ gas. An equilibration run of 5 ns is carried out in the *NPT* ensemble using anisotropic pressure coupling, i.e., only the *z*-direction of the simulation box is allowed to fluctuate. The last 2 ns of the equilibration run are used to calculate the average simulation box dimensions, which are used for an equilibration run of 3 ns in the *NVT* ensemble. Next, production runs of 2 ns are carried out for computing the interfacial tension. In all simulations, 2088 H_2_O molecules are used. Depending on the pressure, the number of H_2_ molecules varied between 64–640. 0–188 Na^+^ and Cl^–^ ions are used, depending on the molality. The exact numbers of species along with the simulation box sizes for all simulations are listed in Table S6 of the Supporting Information. A cutoff radius of 12 Å is used for the short-range LJ and short-range electrostatic energies. Because the system is inhomogeneous, analytic corrections were not used. Instead, long-range LJ and electrostatic interactions are computed using the Particle–Particle Particle–Mesh (PPPM) method.^97,98,101^ For the real and reciprocal space computations for the dispersion part of the PPPM method the desired accuracies are set to 0.0001 and 0.002, respectively. A relative error of 10^–5^ is used for the long-range electrostatic energies. The adequacy of the PPPM method for computing the LJ interactions was recently validated by Salehi et al.^102^ in interfacial MD simulations of deep eutectic solvents with water.

**Figure 1:**
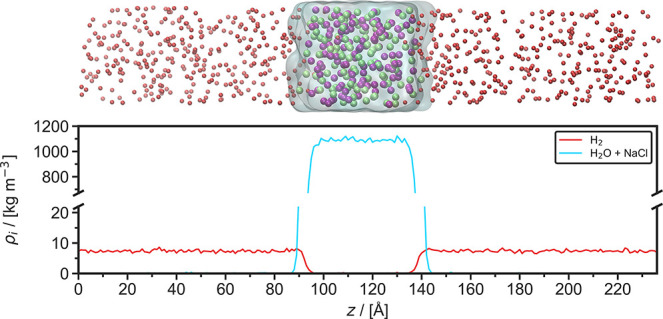
Top: Typical snapshot from a Molecular Dynamics simulation used to calculate the interfacial tension of H_2_ and an aqueous NaCl solution (3 mol NaCl/kg H_2_O) at 343 K and 100 bar. H_2_ molecules are represented by red spheres, Na^+^ and Cl^–^ are represented by purple and green spheres, respectively, H_2_O is represented by the transparent blue surface. Bottom: Density profile in the *z*-direction of H_2_ and the aqueous NaCl solution of the same simulation, averaged over 1 ns. *z* is the direction perpendicular to the interface.

Figure 1 (top panel) shows a typical MD simulation snapshot at *T* = 343 K, *P* = 100 bar, and *m* = 3 mol NaCl/kg H_2_O. The bulk liquid H_2_O phase containing the Na^+^ and Cl^–^ ions, which is shown as the transparent blue surface, occupies a domain of ca. 40 × 40 × 40 Å^3^ in the middle of the simulation box. H_2_ gas is in contact with the liquid phase from both sides, in the *z*-direction. This creates two H_2_/H_2_O interfaces perpendicular to the *z*-direction. The density profile of this system, averaged over 1 ns, is shown in Figure 1 bottom panel.

The interfacial tension γ is calculated from the principal components of the diagonal elements of the stress tensor (*P*_*zz*_, *P*_*xx*_, and *P*_*yy*_):^103^
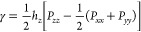
1where *h*_*z*_ is the simulation cell length in the *z*-direction.

#### 2.2.3 Computation of Self-Diffusivities and Viscosities

The scheme used for computing self-diffusivities and viscosities follows from ref (104). Initially, energy minimization of the system is performed, followed by equilibration runs in the *NPT* and *NVT* ensembles for 1–2 ns. Next, production runs in the *NVT* ensemble for 10 ns are carried out. The system consists of 700 H_2_O molecules, 2 H_2_ molecules, and 0–76 Na^+^ and Cl^–^ ions, depending on the molality. The exact numbers of species used for every state point are provided in Table S7 of the Supporting Information. A cutoff radius of 10 Å is used for Lennard-Jones and electrostatic interactions. Analytic tail corrections for energies and pressures are applied.

To compute the self-diffusivities and the shear viscosities, the OCTP plugin^104^ in LAMMPS is used. In this plugin, the Einstein relations are used in combination with the order-*n* algorithm^97^ as implemented by Dubbeldam et al.^105^ Self-diffusivity *D*_*i*_ of species *i* is computed based on the mean-squared displacements using
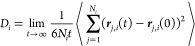
2where ***r***_*j*,*i*(*t*)_ is the position vector of the *j*th molecule of species *i* at time *t* and *N*_*i*_ is the number of molecules of species *i*. All self-diffusivities in this work are corrected for finite-size effects using the Yeh-Hummer equation:^106−108^
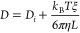
3where *D* is the finite-size corrected self-diffusivity, *T* is the temperature in K, ξ is a dimensionless constant equal to 2.837298, η is the shear viscosity from eq 4, and *L* is the simulation box length. In this work, the finite-size correction magnitude was ca. 5–10% of the computed self-diffusivities.

Shear viscosity η is computed from
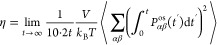
4where
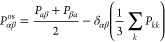
5where *V* is the volume of the system, *k*_B_ is the Boltzmann constant, *P*_*αβ*_^os^ denotes the components of the traceless pressure tensor, δ_*αβ*_ is the Kronecker delta, and ⟨..⟩ indicates an ensemble average. The computation of η does not depend on the size of the system.^109−111^

#### 2.2.4 Computation of Solubilities

Continuous Fractional Component Monte Carlo^78−80^ simulations in the isobaric–isothermal (CFCNPT) ensemble are used to compute solubilities and excess chemical potentials of H_2_ in NaCl solutions. The open-source Brick-CFCMC software^78,112,113^ is used for all simulations. A 10 Å cutoff radius is used for both the LJ and Coulombic interactions. The Ewald summation with a relative precision of 10^–6^ is used for the electrostatics. Analytic tail corrections for energies and pressures are applied.^97^ The infinite dilution excess chemical potential of H_2_ can be computed using a single ”fractional” molecule of H_2_. Fractional molecules have their interactions scaled with a continuous order parameter λ.^78,93^ In CFCNPT simulations, λ ranges from 0 to 1. λ = 0 indicates that the fractional molecule does not interact with the surrounding molecules/atoms (i.e., the fractional molecule behaves as an ideal gas molecule), and λ = 1 corresponds to full interactions. For the specifics regarding the scaling of the interactions the reader is referred elsewhere.^114−116^ To improve the sampling in the λ-space, a biasing weight function (*W*(λ)) is created using the Wang–Landau algorithm.^117,118^ This biasing weight function is used to ensure a flat probability distribution in the λ-space (*p*_obs_(λ)). To compute the probability of occurrence of each λ value, a histogram with 100 bins is used. The Boltzmann averaged probability distributions of λ (*p*(λ)) can be computed using^77,119^
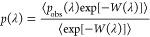
6The Boltzmann sampled probability distribution of λ (*p*(λ)) can be related to the infinite dilution chemical potential (μ^ex^) using^77,78,119^
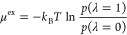
7where *p*(λ = 1) and *p*(λ = 0) are the Boltzmann averaged probability distributions of λ at 1 and 0, respectively.

Five ×10^5^ equilibration cycles, and 5 × 10^5^ production cycles are performed for all simulations. A cycle contains *N* number of trial moves, with *N* being the total number of molecules, with a minimum of 20. The following probabilities are used for selecting the trial moves: 35% translations, 29% rotations, 1% volume changes, 25% λ changes, and 10% reinsertions of the fractional molecules at random locations inside the simulation box. The maximum displacements for molecule translations, volume changes, rotations, and λ changes are adjusted to obtain ca. 50% acceptance. Another method that can be used to compute μ^ex^ is the Widom’s Test Particle Insertion method (WTPI).^96,97,120,121^ In dense fluid phases WTPI yields inaccurate results compared to the CFCMC method as successful insertion of test particles is a highly unlikely event due to the significant potential energy increase in case of overlap with other particles.^78,122^

The solubilities of H_2_ in aqueous NaCl solutions are computed at temperatures in the range (298 to 363) K and at H_2_ partial pressures of 1, 10, 100, 400, and 1000 bar. At H_2_ partial pressures of 1 and 10 bar, the H_2_ solubilities are computed using Henry coefficients (*H*):
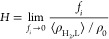
8where *f*_*i*_ is the fugacity of H_2_ in the gas phase, ⟨ρ_H_2_,L_⟩ is the average number density of H_2_ in the aqueous solution in units of 1/m^3^, and ρ_0_ is a reference number density in the same unit as ρ_H_2_,L_ (set to 1 molecule per m^3^).^119^ At H_2_ partial pressures of 1 and 10 bar, the fugacity coefficient of H_2_ is assumed to be 1 (i.e., the fugacity of H_2_ is equal to the partial pressure of H_2_). The validity of eq 8 at H_2_ partial pressures of 1–100 bar is discussed in Figure S3 of the Supporting Information. From the MC simulations, *H* can be computed using^119^
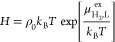
9where μ_H_2_,L_^ex^ is the infinite dilution chemical potential of H_2_ in the aqueous solution. A single fractional molecule of H_2_, 300 H_2_O molecules, and varying number of NaCl molecules depending on the molality (ranging from 0 to 6 mol NaCl/kg H_2_O) are used to compute μ_H_2_,L_^ex^. The exact numbers of ions used for each molality are listed in Table S8 of the Supporting Information. To calculate solubilities of H_2_ in aqueous NaCl solutions at pressures of 100, 400, and 1000 bar, the chemical potentials of H_2_ in the liquid and in the gas phase are equated at constant pressure and temperature. The chemical potential of H_2_ in the gas phase (μ_H_2_, G_) is equal to^97^
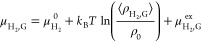
10where μ_H_2__^0^ is the reference state of the chemical potential, ⟨ρ_H_2_,G_⟩ is the average number density of H_2_ in the gas phase in units of 1/m^3^, and μ_H_2_,G_^ex^ is the excess chemical potential of H_2_ in the gas phase. At pressures above 100 bar and at temperatures between (298 to 363) K, the gas phase contains very few H_2_O molecules (a H_2_O mole fraction below 0.01).^93^ μ_H_2_,G_^ex^ is calculated in separate CFCNPT simulations, containing a single fractional molecule of H_2_, and 300 H_2_ molecules in the gas phase. At conditions where the gas phase is nonideal and the H_2_O content in the gas phase is not negligible (i.e., temperatures above 363 K and pressures above 100 bar), Gibbs ensemble simulations can be performed to simulate the gas and the liquid phase simultaneously.^93^ These simulations are beyond the scope of this work. The chemical potential of H_2_ in the liquid phase (μ_H_2_, L_) is equal to^97^
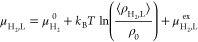
11⟨ρ_H_2_,L_⟩ can be computed by equating eq 10 and eq 11. The mole fractions of H_2_ (*x*_H_2__) in aqueous NaCl solutions are computed using
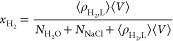
12where ⟨*V*⟩ is the average volume of the simulation box, computed in the CFCNPT ensemble. *N*_H_2_O_ and *N*_NaCl_ are the numbers of H_2_O and NaCl molecules in the simulation box, respectively. For each condition (concentration, temperature, and pressure), 20 independent simulations are performed. These 20 simulations are divided into 5 blocks from which the Boltzmann sampled probability distributions of λ (*p*(λ)) are averaged. The averaged distributions (*p*(λ)) of all blocks are used to compute mean values and standard deviations for the excess chemical potentials and solubilities of H_2_.

## 3 Results and Discussion

### 3.1 Interfacial Tensions

**Figure 2:**
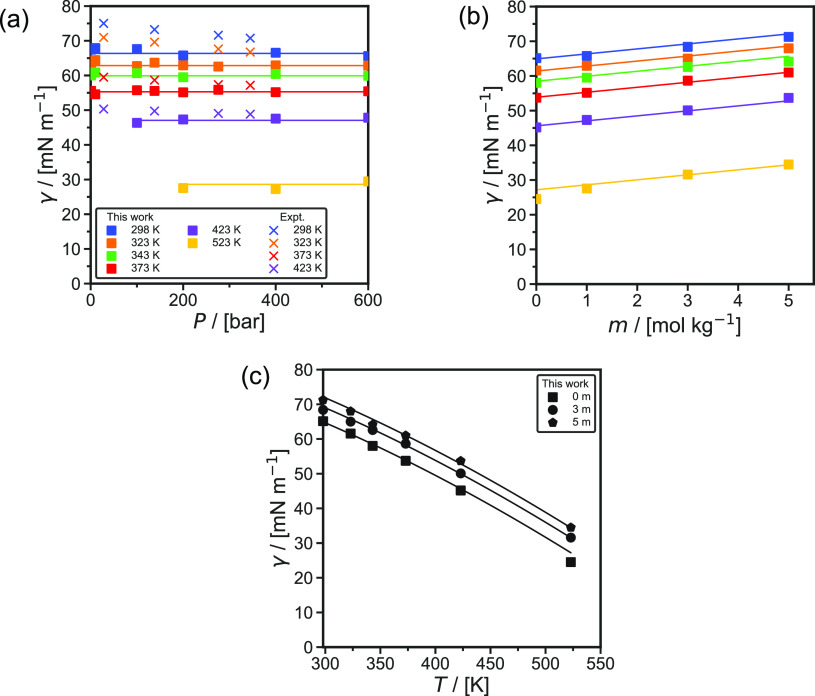
MD results of interfacial tension γ of H_2_ and aqueous NaCl solutions using the NaCl Madrid-2019^81^ force fields, the TIP4*P*/2005^69^ H_2_O force field, and the Vrabec^64^ H_2_ force field (a) as functions of pressure *P* for temperatures in a range of (298 to 523) K with a molality *m* of 1 mol NaCl/kg H_2_O in combination with the experimental results of Hosseini et al.,^27^ (b) as functions of molality at a pressure of 200 bar of the solution for similar temperatures, and (c) as functions of temperature at a pressure of 200 bar and molalities of (0 to 6) mol NaCl/kg H_2_O. The statistical uncertainties are comparable to or smaller than the symbols and can be found in Table S9 of the Supporting Information. The error bars have been omitted for clarity. The solid lines represent fits using eq 13 for temperatures in the range of (298 to 523) K.

Figure 2 shows the computed interfacial tensions of H_2_/H_2_O/NaCl as a function of pressure (Figure 2a), molality (Figure 2b) at temperatures in the range of (298 to 523) K, and as a function of temperature (Figure 2c) for molalities in the range of (0 to 6) mol NaCl/kg H_2_O. Tabulated raw data of the interfacial tension along with their statistical uncertainties are listed in Table S9 of the Supporting Information. Figure 2a and 2c also show the available experimental data from Hosseini et al.^27^ For the whole range of conditions a close agreement with the experimental results is found. The MD results differ on average 6.4% from the experimental values. The simulations at 523 K cannot be directly validated due to lack of experimental data.

The interfacial tensions computed in this work are fitted to an engineering equation:
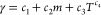
13where *c*_1_, *c*_2_, *c*_3_, and *c*_4_ are fitting parameters, which are listed in Table 2. Equation 13 is valid for temperatures, pressures, and molalities of (298 to 523) K, (1 to 600) bar, and (0 to 6) mol NaCl/kg H_2_O, respectively. The results of this engineering equation are shown as solid lines in Figure 2. Equation 13 is a very good fit to MD results, and can be used for calculating values at a specific combination of conditions in a fast and reliable way.

**Table 2:** Parameters of eq 13 for Predicting Interfacial Tension of H_2_ in Contact with Aqueous NaCl Solutions^a^

*c*_1_/[mN/m]	89.6
*c*_2_/[(mN·kg_H_2_O_)/(m·mol_NaCl_)]	1.44
*c*_3_/[mN/(m·K^1.65^)]	–2.04 × 10_–3_
*c*_4_/[−]	1.65

a Values obtained using the NaCl Madrid-2019^81^ force fields, TIP4P/2005^69^ H_2_O force field, and Vrabec^64^ H_2_ force field. These parameters are valid for NaCl molalities of (0 to 6) mol NaCl/kg H_2_O, temperatures of (298 to 523) K, and pressures of (1 to 600) bar.

As shown in Figure 2a, no significant pressure dependence of interfacial tension is observed. The data of experimental studies also show no significant or small pressure dependences.^22,25,27,28^ In particular, Higgs et al.^28^ did not observe a significant pressure dependence for H_2_ in contact with aqueous NaCl solutions. Other studies observed a small decrease in interfacial tension of H_2_ and pure H_2_O^22,25,27^ and H_2_ and aqueous (NaCl+KCl) solutions.^27^ Interestingly, the pressure dependence of H_2_/H_2_O interfacial tension is small compared to the CO_2_/H_2_O^123−125^ and CH_4_/H_2_O^51,126,127^ systems. This is because the interfacial tension is related to the density difference between the two phases.^16^ The change in density difference between H_2_ and H_2_O is very small at varying pressures because the density of H_2_ is very low in comparison to H_2_O, and H_2_O is almost incompressible at these pressures.

As shown in Figure 2b, the interfacial tension increases linearly with solution molalities, in agreement with the available experimental data.^27^ This behavior is also observed for other gases such as CO_2_ and CH_4_.^128−130^ This increase is mainly due to the increased density of saline H_2_O compared to pure H_2_O as well as the arrangement of cations and anions at the interface.^129,131−135^ The hydrogen bond network of H_2_O is strengthened by cations,^132−134^ while anions cause the opposite effect.^132−134^ Therefore, cations are absorbed into the bulk phase while anions are depleted from the bulk phase. This phenomenon can be observed in Figure S2 of the Supporting Information, where it is shown that the number density of Cl^–^ ions at the interface is higher than Na^+^ ions, and Na^+^ ions are drawn into the bulk phase. The strengthening of the hydrogen bond network of H_2_O leads to an increase in interfacial tension.^129,131^

In Figure 2c, a nonlinear decrease of interfacial tension with temperature can be observed. This is in line with the experimental data of Chow et al.^25^ In sharp contrast, Hosseini et al.^27^ reported a linear decrease of interfacial tension with temperature. The fact that interfacial tension depends nonlinearly on the density difference between the two phases in contact^16^ combined with the nonlinear effect of temperature on the density difference between H_2_ and H_2_O, results in the expectation that the interfacial tension is also nonlinearly related to temperature. Therefore, the observed nonlinear relationship between interfacial tension and temperature in our results is expected.

### 3.2 Viscosities and Densities

**Figure 3:**
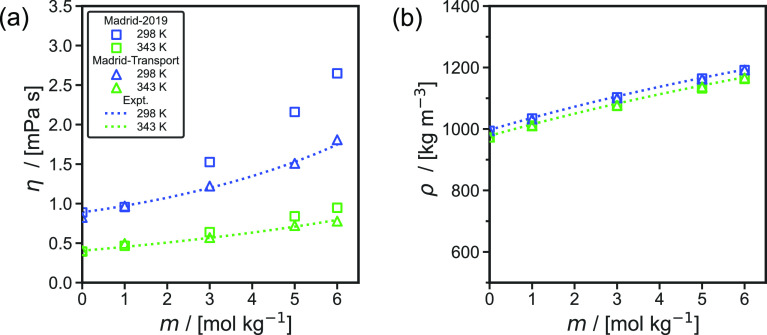
Computed (a) viscosities η and (b) densities ρ of aqueous NaCl solutions as a function of molality *m* (mol NaCl/kg H_2_O) at a pressure of 1 bar and temperatures of 298 and 343 K. The fit to the experimental data is created by (a) Laliberté^136^ and (b) Laliberté and Cooper.^137^ The statistical uncertainties can be found in Table S10 of the Supporting Information. The error bars are smaller or comparable to the symbol size and have been omitted for clarity.

Figure 3 shows the computed densities and viscosities of aqueous NaCl solutions as functions of NaCl molalities at 298 and 343 K. The densities are computed from the average volume calculated from the *NPT* ensemble. Equation 4 is used to compute the viscosities. Densities and viscosities of aqueous NaCl solutions have a weaker dependence on pressure (in the range of 0–1000 bar) compared to temperature and NaCl molalities. Figures S4 and S5 in the Supporting Information show the densities and the viscosities as functions of pressure. The results for the Madrid-2019^81^ and the Madrid-Transport^77,82^ NaCl force fields are shown in Figure 3. Laliberté^136^ and Laliberté and Cooper^137^ developed models on the basis of experimental data for viscosities and densities, respectively. These fits are shown in Figure 3. The raw data of these properties, as well as their statistical uncertainties, are listed in Table S10 of the Supporting Information. Both the Madrid-2019^81^ and Madrid-Transport^77,82^ capture the experimental data of density very accurately (within 1%). As shown in Figure 3a, the Madrid-Transport^77,82^ force field yields a better agreement with the experimental data of viscosity compared to the Madrid-2019^81^ force field. The discrepancy between the two force fields starts at molalities above 2 mol NaCl/kg H_2_O. The viscosities computed using the Madrid-2019 force field deviate on average ca. 20% from the experimental data, while this deviation is only ca. 3% when the Madrid-Transport force field is used. Based on the excellent performance of Madrid-Transport in reproducing experimental viscosities, which is necessary for reliable diffusivity predictions,^138^ only this force field was used for computing the self-diffusivities of H_2_ in NaCl solutions.

### 3.3 Self-Diffusivities of H_2_

**Figure 4:**
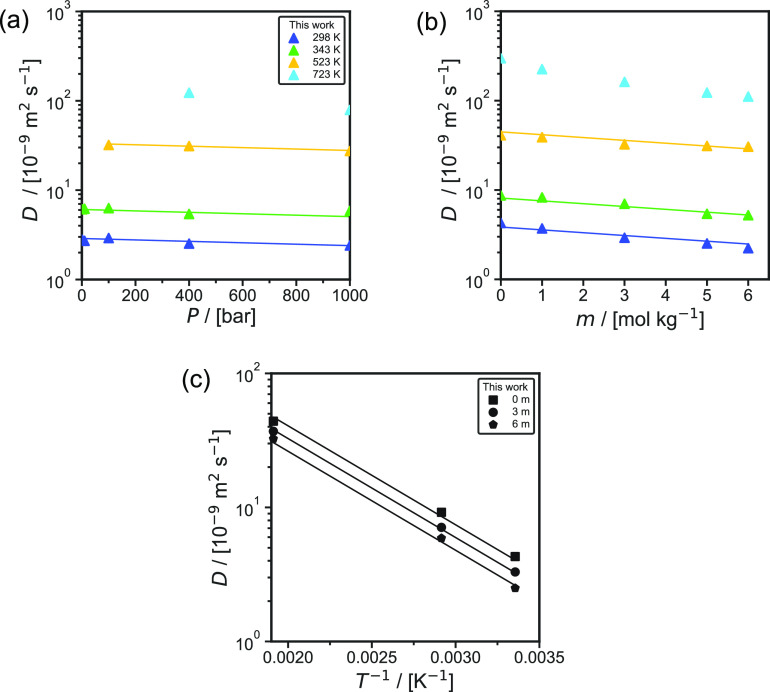
Computed finite-size corrected self-diffusivities (*D*) of H_2_ in aqueous NaCl solutions (a) with a molality (*m*) of 5 mol NaCl/kg H_2_O solution as a function of pressure *P* for temperatures of (298 to 723) K, (b) at a pressure of 400 bar as a function of *m* of the solution for the similar temperatures, and (c) as a function of the reciprocal temperature at a pressure of 100 bar. The results are obtained using the NaCl Madrid-Transport^77,82^ force fields, TIP4P/2005^69^ H_2_O force field, and Vrabec^64^ H_2_ force field. The statistical uncertainties are comparable to or smaller than the symbols and can be found in Table S11 of the Supporting Information. The solid lines are fits calculated using eq 14 for temperatures of (298 to 523) K. Data points at a temperature of 723 K and a pressure of 400 bar are excluded from the fit because H_2_O is supercritical at these conditions.

Figure 4 shows the computed finite-size corrected self-diffusivities of H_2_ in aqueous NaCl solutions as a function of (a) pressure, (b) NaCl molality, and (c) temperature. These simulation are performed with the Madrid-Transport^77,82^ force field for NaCl, the TIP4P/2005^69^ H_2_O, and the Vrabec^64^ force field for H_2_. All the self-diffusivities of H_2_ computed in this work are listed in Tables S11 and S12 of the Supporting Information. Simulations using the Madrid-2019^81^ NaCl force field are performed for comparison. These data are shown in Table S12 of the Supporting Information.

The self-diffusivities of H_2_ shown in Figure 4 are fitted to an engineering correlation:
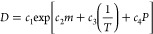
14where *c*_1_, *c*_2_, *c*_3_, and *c*_4_ are fitting parameters, which are listed in Table 3. As shown in Figure 4, this correlation provides an excellent fit for the MD results. Note that eq 14 is only valid for conditions at which H_2_O is in the liquid phase, and therefore, data points at temperatures of 723 K or higher are excluded as the solution is in the supercritical phase. In this work, self-diffusivities for temperatures of 723 K and pressures lower than 400 bar are not calculated, because at those conditions, H_2_O is in the gas phase. Equation 14 is an empirical model.

**Table 3:** Parameters of eq 14 for Predicting the Computed Finite-Size Corrected Self-Diffusivities *D* of H_2_ in Aqueous NaCl Solutions^a^

*c*_1_/[m^2^/s]	1.24 × 10^–6^
*c*_2_/[(mol_NaCl_/kg_H_2_O_)^−1^]	–7.29 × 10^–2^
*c*_3_/[K]	–1.70 × 10^3^
*c*_4_/[bar^–1^]	–1.84 × 10^–4^

a Values obtained using the NaCl Madrid-Transport^77,82^ force fields, the TIP4P/2005^69^ H_2_O force field, and the Vrabec^64^ H_2_ force field. These parameters are valid for NaCl molalities of (0 to 6) mol NaCl/kg H_2_O, temperatures of (298 to 523) K, and pressures of (1 to 1000) bar. Note that eq 14 should only be used at conditions in which water is in the liquid state.

In Figure 4a a weak pressure dependence of the self-diffusivities of H_2_ is observed. The logarithm of the self-diffusivities decays linearly with respect to variations in pressure, similarly to what is reported by Tsimpanogiannis et al.^84^ The pressure dependence of the self-diffusivities of H_2_ is more significant at 723 K (Figure 4a) as the solution is more compressible at these conditions. As shown in Figure 4b, the logarithm of the self-diffusivities is also found to decay linearly with respect to variation in the NaCl molalities. Laliberté^136^ has shown that the viscosities of aqueous NaCl solutions increase exponentially with respect to NaCl molalities. As the self-diffusivities of gases dissolved in liquids are inversely proportional to the viscosities of the solution,^16,138^ the self-diffusivities of H_2_ are expected to decay exponentially with respect to the NaCl molalities. The computed self-diffusivities of H_2_ follow an Arrhenius-type^97^ relation with respect to variations in temperature () as shown in Figure 4c. This behavior is also observed in literature for gases (e.g., O_2_, H_2_) dissolved in aqueous solvents.^60,86,139−141^

### 3.4 Solubilities

**Figure 5:**
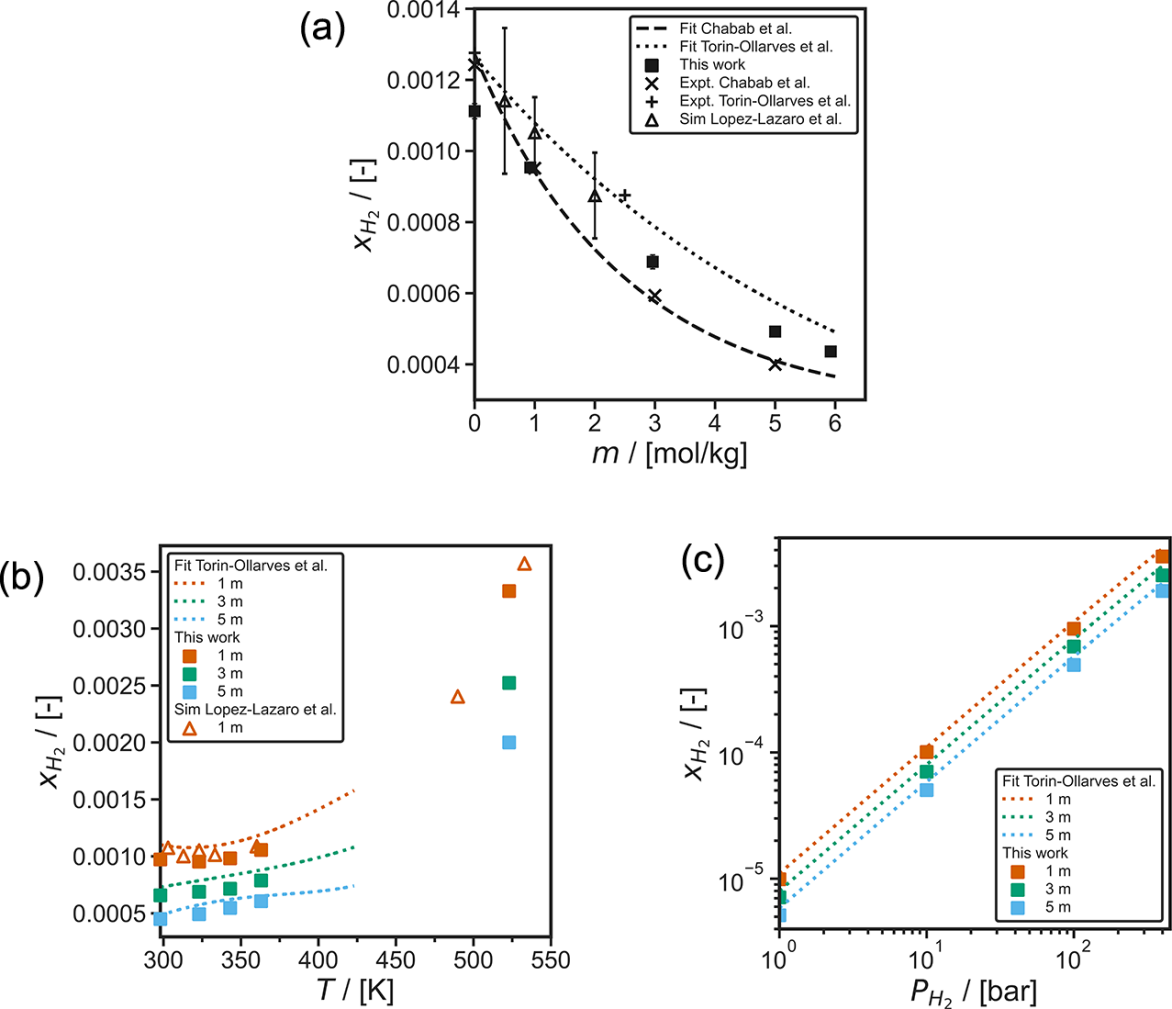
Computed solubilities of H_2_ in aqueous NaCl solutions using the NaCl Madrid-2019 force field,^81^ TIP4P/2005^69^ H_2_O force field, and Marx^63^ H_2_ force field as functions of (a) NaCl molality (*m*) in units of mol NaCl/kg H_2_O for a H_2_ partial pressure of 100 bar, at 323 K, (b) temperature for a H_2_ partial pressure of 100 bar, and (c) H_2_ partial pressure at 323 K. The dashed lines represent the experimental correlation provided by Torín-Ollarves and Trusler,^32^ and the dotted lines represent the experimental correlation results of Chabab et al.^31^ The experimental data of Chabab et al.,^31^ and Torín-Ollarves and Trusler,^32^ and the simulation results of Lopez-Lazaro et al.^74^ (converted from Henry constants) are also shown. The error bars of the simulations of this work are comparable (Figure 5a) or smaller than (Figure 5b and 5c) the symbol size.

In Figure 5, the solubilities of H_2_ computed using CFCMC are shown as a function of (a) NaCl molality, (b) temperature, and (c) pressure. The computed solubilities are compared to the experimental measurements of Chabab et al.,^31^ Torín-Ollarves and Trusler,^32^ and their corresponding experimental correlations (also shown in Figure 5). The correlation of Torín-Ollarves and Trusler^32^ is based on the experimental measurements for NaCl molalities of 0 and 2.5 mol NaCl/kg H_2_O, the experimental data by Wiebe and Gaddy,^142^ Wiebe et al.,^143^ Kling and Maurer,^144^ and Choudhary et al.^145^ for H_2_ solubility in pure H_2_O, and the experiments by Crozier and Yamamoto^37^ and Gordon et al.^38^ for solubility of H_2_ in saline solutions. Chabab et al.^31^ provide an extensive experimental data set and a correlation for H_2_ solubilities at temperatures of (323 to 373) K, and NaCl molalities in the range of (0 to 5) mol/kg H_2_O. Lopez-Lazaro et al.^74^ obtained the Henry constants of H_2_ in aqueous NaCl solutions for molalities up to 2 mol NaCl/kg H_2_O using excess chemical potentials computed using the WTPI method.^96,97,120,121^ Using the Henry constants reported by Lopez-Lazaro et al.,^74^ the solubilities of H_2_ at a partial pressure of 100 bar are computed and shown in Figures 5a and 5b. The simulations of Lopez-Lazaro et al.^74^ show large error bars (ca. 10–20%). This may be due to the use of the WTPI method,^96,97,120,121^ which requires a large number of MC cycles to obtain low standard deviations for excess chemical potentials in the liquid phase.^74,78^ The solubilities computed in this work using CFCMC simulations^79,80,112,113^ have uncertainties of less than 5%.

Figure 5a shows the decrease of the solubilities of H_2_ at increasing molalities of NaCl (i.e., salting-out effect). The salting-out of nonpolar gases (e.g., H_2_, O_2_, and CO_2_) in the presence of salts such as NaCl, KCl, and KOH is a well observed phenomenon.^57,77,135^ As shown in Figure 5a, the models by Torín-Ollarves and Trusler^32^ and Chabab et al.^31^ agree for H_2_ solubilities in pure H_2_O and at NaCl molalities below 0.5 mol NaCl/kg H_2_O. For NaCl concentrations higher than 0.5 mol NaCl/kg H_2_O the two models predict different H_2_ solubilities. The salting-out effect of H_2_ observed in this work using the Madrid-2019^81^ Na^+^ and Cl^–^, TIP4P/2005^69^ H_2_O, and the Marx^63^ H_2_ force fields shows better agreement to the salting-out effect observed by Torín-Ollarves and Trusler^32^ as shown in Figure 5a. The correlation of Torín-Ollarves and Trusler^32^ also shows agreement with our simulations at H_2_ partial pressures ranging from (1 to 400) bar in the temperature range (298 to 363) K, as shown in Figures 5b and 5c. Our simulation results also agree with the MC simulations by Lopez-Lazaro et al.^74^ both for different NaCl molalities, and for different temperatures in the range of (298 to 523) K, even though the choice of the force fields for Na^+^, Cl^–^, and H_2_ is different. Lopez-Lazaro et al.^74^ have used the OPLS force field^146^ for Na^+^, and Cl^–^, combined with the model by Darkim et al.^147^ for H_2_.

In Table S13 of the Supporting Information, we provide an extensive database for solubilities of H_2_ at temperatures of (298 to 363) K, H_2_ partial pressures of (1 to 1000) bar, and at NaCl molalities of (0 to 6) mol/kg H_2_O. The solubilities of H_2_ at partial pressures up to 100 bar are computed for a wider temperature range, i.e., (298 to 523) K. These data can be further used to test and train existing machine-learning models^33^ or equations of state^148^ for predicting H_2_ solubilities in saline solutions at conditions relevant to geological H_2_ storage.

## 4 Conclusions

Molecular simulations are used to compute (a) interfacial tensions of H_2_ and aqueous NaCl solutions for temperatures, pressures, and molalities of (298 to 523) K, (1 to 600) bar, and (0 to 6) mol NaCl/kg H_2_O, respectively, (b) self-diffusivities of H_2_ in aqueous NaCl solutions for temperatures, pressures, and molalities of (298 to 723) K, (1 to 1000) bar and (0 to 6) mol NaCl/kg H_2_O, respectively, and (c) solubilities of H_2_ in aqueous NaCl solutions for temperatures, pressures, and molalities of (298 to 363) K, (1 to 1000) bar, and (0 to 6) mol NaCl/kg H_2_O, respectively. The simulations for computing H_2_ self-diffusivities are also used to yield predictions for densities and viscosities of the NaCl solutions. The interfacial tensions and self-diffusivities are computed using MD simulations, and the solubilities are computed using CFCMC simulations. The H_2_O TIP4P/2005^69^ force field, the NaCl Madrid-2019^81^ force fields, and H_2_ Vrabec^64^ and Marx^63^ force fields are used. In addition, a modified version of the Madrid-2019 force field (i.e., the Madrid-Transport^77,82^ force field) is used, which is optimized for transport properties of aqueous solutions. Our results are validated against the available experimental data, models, and simulations. Excellent agreement between the results and experimental data is found with deviations smaller than 10% for the vast majority of the data points. The results of the NaCl Madrid-Transport force field were in better agreement with experimental data for transport properties, while the Madrid-2019 force field was sufficiently accurate for interfacial tensions and solubilities. The new data are used to develop engineering equations for interfacial tension and self-diffusion capturing the effect of pressure, temperature, and solution molality.

## Supporting Information Available

Solubilities of H_2_ in pure water; force field parameters; additional info MD and CFCMC simulations; raw data MD simulations for interfacial tensions; density and viscosity data; raw data MD simulations for self-diffusivities; raw data CFCMC simulations; solubilities of H_2_ in pure water; density profile of Na^+^ and Cl^–^ of a MD simulation to calculate interfacial tensions; solubilities of H_2_ in aqueous NaCl solution at H_2_ partial pressures of 1–100 bar; densities of NaCl solutions; viscosities of NaCl solutions (PDF)

## References

[ref1] World Energy Outlook 2021. 2021; www.iea.org/weo (accessed: 10/10/2022).

[ref2] United Nations, The Paris Agreement. 2015; https://treaties.un.org/pages/ViewDetails.aspx?src=TREATY&mtdsg_no=XXVII-7-d&chapter=27&clang=_en (accessed: 10/10/2022).

[ref3] Johnston B.; Mayo M. C.; Khare A. Hydrogen: the energy source for the 21st century. Technovation 2005, 25, 569–585. 10.1016/j.technovation.2003.11.005.

[ref4] Mahlia T.; Saktisahdan T.; Jannifar A.; Hasan M.; Matseelar H. A review of available methods and development on energy storage; technology update. Renewable Sustainable Energy Rev. 2014, 33, 532–545. 10.1016/j.rser.2014.01.068.

[ref5] Kovač A.; Paranos M.; Marciuš D. Hydrogen in energy transition: A review. Int. J. Hydrogen Energy 2021, 46, 10016–10035. 10.1016/j.ijhydene.2020.11.256.

[ref6] Carden P. O.; Paterson L. Physical, chemical and energy aspects of underground hydrogen storage. Int. J. Hydrogen Energy 1979, 4, 559–569. 10.1016/0360-3199(79)90083-1.

[ref7] Zivar D.; Kumar S.; Foroozesh J. Underground hydrogen storage: A comprehensive review. Int. J. Hydrogen Energy 2021, 46, 23436–23462. 10.1016/j.ijhydene.2020.08.138.

[ref8] Tarkowski R. Underground hydrogen storage: Characteristics and prospects. Renewable Sustainable Energy Rev. 2019, 105, 86–94. 10.1016/j.rser.2019.01.051.

[ref9] Hashemi L.; Blunt M.; Hajibeygi H. Pore-scale modelling and sensitivity analyses of hydrogen-brine multiphase flow in geological porous media. Sci. Rep. 2021, 11, 1–13. 10.1038/s41598-021-87490-7. 33863943 PMC8052453

[ref10] Ursúa A.; Gandía L. M.; Sanchis P. Hydrogen production from water electrolysis: Current status and future trends. Proc. IEEE 2012, 100, 410–426. 10.1109/JPROC.2011.2156750.

[ref11] Grigoriev S. A.; Fateev V. N.; Bessarabov D. G.; Millet P. Current status, research trends, and challenges in water electrolysis science and technology. Int. J. Hydrogen Energy 2020, 45, 26036–26058. 10.1016/j.ijhydene.2020.03.109.

[ref12] Pan B.; Yin X.; Ju Y.; Iglauer S. Underground hydrogen storage: Influencing parameters and future outlook. Adv. Colloid Interface Sci. 2021, 294, 102473 10.1016/j.cis.2021.102473. 34229179

[ref13] Haug P.; Koj M.; Turek T. Influence of process conditions on gas purity in alkaline water electrolysis. Int. J. Hydrogen Energy 2017, 42, 9406–9418. 10.1016/j.ijhydene.2016.12.111.

[ref14] Haug P.; Kreitz B.; Koj M.; Turek T. Process modelling of an alkaline water electrolyzer. Int. J. Hydrogen Energy 2017, 42, 15689–15707. 10.1016/j.ijhydene.2017.05.031.

[ref15] Zarghami A.; Deen N.; Vreman A. CFD modeling of multiphase flow in an alkaline water electrolyzer. Chem. Eng. Sci. 2020, 227, 115926 10.1016/j.ces.2020.115926.

[ref16] Poling B. E.; Prausnitz J. M.; O’Connell J. P. Properties of gases and liquids, 5th ed.; McGraw-Hill Education: New York, 2001.

[ref17] Zoulias E.; Varkaraki E.; Lymberopoulos N.; Christodoulou C. N.; Karagiorgis G. N. A review on water electrolysis. Tcjst 2004, 4, 41–71.

[ref18] Hnát J.; Paidar M.; Bouzek K.; Iulianelli A.; Basile A. Current Trends and Future Developments on (Bio-) Membranes; Elsevier: Amsterdam, 2020.

[ref19] Hauch A.; Ebbesen S. D.; Jensen S. H.; Mogensen M. Highly efficient high temperature electrolysis. J. Mater. Chem. 2008, 18, 2331–2340. 10.1039/b718822f.

[ref20] Todd D.; Schwager M.; Mérida W. Thermodynamics of high-temperature, high-pressure water electrolysis. J. Power Sources 2014, 269, 424–429. 10.1016/j.jpowsour.2014.06.144.

[ref21] Holm T.; Borsboom-Hanson T.; Herrera O. E.; Mérida W. Hydrogen costs from water electrolysis at high temperature and pressure. Energy Convers. Manage. 2021, 237, 114106 10.1016/j.enconman.2021.114106.

[ref22] Slowinski E. J.; Gates E. E.; Waring C. E. The effect of pressure on the surface tensions of liquids. J. Phys. Chem. 1957, 61, 808–810. 10.1021/j150552a028.

[ref23] Braun L. Über die Absorption von Stickstoff und von Wasserstoff in wässerigen Lösungen verschieden dissociierter Stoffe. Z. Phys. Chem. 1900, 33U, 721–739. 10.1515/zpch-1900-3349.

[ref24] Gertz K.; Loeschcke H. Bestimmung der Diffusions-Koeffizienten von H_2_, O_2_, N_2_, und He in Wasser und Blutserum bei konstant gehaltener Konvektion. Z. Naturforsch. B 1954, 9, 1–9. 10.1515/znb-1954-0102.

[ref25] Chow Y. T.; Maitland G. C.; Trusler J. P. Interfacial tensions of (H_2_O + H_2_) and (H_2_O + CO_2_ + H_2_) systems at temperatures of (298–448) K and pressures up to 45 MPa. Fluid Phase Equilib. 2018, 475, 37–44. 10.1016/j.fluid.2018.07.022.

[ref26] Massoudi R.; King A. D. Jr Effect of pressure on the surface tension of water. Adsorption of low molecular weight gases on water at 25 °C. J. Phys. Chem. 1974, 78, 2262–2266. 10.1021/j100615a017.

[ref27] Hosseini M.; Fahimpour J.; Ali M.; Keshavarz A.; Iglauer S. H_2_-brine interfacial tension as a function of salinity, temperature, and pressure; implications for hydrogen geo-storage. J. Pet. Sci. Eng. 2022, 213, 110441 10.1016/j.petrol.2022.110441.

[ref28] Higgs S.; Da Wang Y.; Sun C.; Ennis-King J.; Jackson S. J.; Armstrong R. T.; Mostaghimi P. In-situ hydrogen wettability characterisation for underground hydrogen storage. Int. J. Hydrogen Energy 2022, 47, 13062–13075. 10.1016/j.ijhydene.2022.02.022.

[ref29] Richards T. W.; Carver E. K. A critical study of the capillary rise method of determining surface tension, with data for water, benzene, toluene, chloroform, carbon tetrachloride, ether and dimethyl aniline.[second paper.]. J. Am. Chem. Soc. 1921, 43, 827–847. 10.1021/ja01437a012.

[ref30] Drelich J.; Fang C.; White C. Measurement of interfacial tension in fluid-fluid systems. Encycl. Surf. Colloid Sci. 2002, 3, 3158–3163.

[ref31] Chabab S.; Théveneau P.; Coquelet C.; Corvisier J.; Paricaud P. Measurements and predictive models of high-pressure H_2_ solubility in brine (H_2_O+NaCl) for underground hydrogen storage application. Int. J. Hydrogen Energy 2020, 45, 32206–32220. 10.1016/j.ijhydene.2020.08.192.

[ref32] Torín-Ollarves G. A.; Trusler J. M. Solubility of hydrogen in sodium chloride brine at high pressures. Fluid Phase Equilib. 2021, 539, 113025 10.1016/j.fluid.2021.113025.

[ref33] Ansari S.; Safaei-Farouji M.; Atashrouz S.; Abedi A.; Hemmati-Sarapardeh A.; Mohaddespour A. Prediction of hydrogen solubility in aqueous solutions: Comparison of equations of state and advanced machine learning-metaheuristic approaches. Int. J. Hydrogen Energy 2022, 47, 37724 10.1016/j.ijhydene.2022.08.288.

[ref34] Steiner P. Ueber die Absorption des Wasserstoffs im Wasser und in wässerigen Lösungen. Ann. Phys. 1894, 288, 275–299. 10.1002/andp.18942880605.

[ref35] Knopp W. Über die Löslichkeitsbeeinflussung von Wasserstoff und Stickoxydul in wässerigen Lösungen verschieden dissoziierter Stoffe. Z. Phys. Chem. 1904, 48U, 97–108. 10.1515/zpch-1904-4806.

[ref36] Gerecke J.; Bittrich H. The solubility of H_2_, CO_2_ and NH_3_ in an aqueous electrolyte solution. Wiss. Z. Tec.h Hochsch. Chem. Carl Schorlemmer Leuna Merseburg 1971, 13, 115–122.

[ref37] Crozier T. E.; Yamamoto S. Solubility of Hydrogen in Water, Seawater, and NaCl Solutions. J. Chem. Eng. Data 1974, 19, 242–244. 10.1021/je60062a007.

[ref38] Gordon L. I.; Cohen Y.; Standley D. R. The solubility of molecular hydrogen in seawater. Deep-Sea Res. 1977, 24, 937–941. 10.1016/0146-6291(77)90563-X.

[ref39] Winkelmann J. Landolt-Bornstein: Numerical Data and Functional Relationships in Science and Technology, Group IV, 1st ed.; Springer-Verlag: New York, 2007; Vol. 15A.

[ref40] Baird M. H. I.; Davidson J. F. Annular jets-II: Gas absorption. Chem. Eng. Sci. 1962, 17, 473–480. 10.1016/0009-2509(62)85016-7.

[ref41] Houghton G.; Wise D. L. The diffusion coefficients of ten slightly soluble gases in water at 10–60° C. Chem. Eng. Sci. 1966, 21, 999–1010. 10.1016/0009-2509(66)85096-0.

[ref42] Akgerman A.; Gainer J. L. Predicting gas-liquid diffusivities. J. Chem. Eng. Data 1972, 17, 372–377. 10.1021/je60054a008.

[ref43] Himmelblau D. M. Diffusion of dissolved gases in liquids. Chem. Rev. 1964, 64, 527–550. 10.1021/cr60231a002.

[ref44] Verhallen P. T. H. M.; Oomen L. J. P.; Elsen A. J. J. M.; Kruger J.; Fortuin J. M. H. The diffusion coefficients of helium, hydrogen, oxygen and nitrogen in water determined from the permeability of a stagnant liquid layer in the quasi-s. Chem. Eng. Sci. 1984, 39, 1535–1541. 10.1016/0009-2509(84)80082-2.

[ref45] Jähne B.; Heinz G.; Dietrich W. Measurement of the diffusion coefficients of sparingly soluble gases in water. J. Geophys. Res.: Oceans 1987, 92, 10767–10776. 10.1029/JC092iC10p10767.

[ref46] De Blok W. J.; Fortuin J. M. H. Method for determining diffusion coefficients of slightly soluble gases in liquids. Chem. Eng. Sci. 1981, 36, 1687–1694. 10.1016/0009-2509(81)80014-0.

[ref47] Nielsen L. C.; Bourg I. C.; Sposito G. Predicting CO_2_-water interfacial tension under pressure and temperature conditions of geologic CO_2_ storage. Geochim. Cosmochim. Acta 2012, 81, 28–38. 10.1016/j.gca.2011.12.018.

[ref48] Li X.; Ross D. A.; Trusler J. P. M.; Maitland G. C.; Boek E. S. Molecular dynamics simulations of CO_2_ and brine interfacial tension at high temperatures and pressures. J. Phys. Chem. B 2013, 117, 5647–5652. 10.1021/jp309730m. 23537183

[ref49] Liu Y.; Lafitte T.; Panagiotopoulos A. Z.; Debenedetti P. G. Simulations of vapor-liquid phase equilibrium and interfacial tension in the CO_2_-H_2_O-NaCl system. AIChE J. 2013, 59, 3514–3522. 10.1002/aic.14042.

[ref50] Tsuji S.; Liang Y.; Kunieda M.; Takahashi S.; Matsuoka T. Molecular Dynamics Simulations of the CO_2_-Water-silica Interfacial Systems. Energy Procedia 2013, 37, 5435–5442. 10.1016/j.egypro.2013.06.462.

[ref51] Knauer S.; Schenk M. R.; Koddermann T.; Reith D.; Jaeger P. Interfacial tension and related properties of ionic liquids in CH_4_ and CO_2_ at elevated pressures: experimental data and molecular dynamics simulation. J. Chem. Eng. Data 2017, 62, 2234–2243. 10.1021/acs.jced.6b00751.

[ref52] Hosseinzadeh Dehaghani Y.; Assareh M.; Feyzi F. Simultaneous Prediction of Equilibrium, Interfacial, and Transport Properties of CO_2_-Brine Systems Using Molecular Dynamics Simulation: Applications to CO_2_ Storage. Ind. Eng. Chem. Res. 2022, 61, 15390–15406. 10.1021/acs.iecr.2c02249.

[ref53] Zhao L.; Ji J.; Tao L.; Lin S. Ionic effects on supercritical CO_2_-brine interfacial tensions: Molecular dynamics simulations and a universal correlation with ionic strength, temperature, and pressure. Langmuir 2016, 32, 9188–9196. 10.1021/acs.langmuir.6b02485. 27564433

[ref54] Yang Y.; Narayanan Nair A. K.; Sun S. Molecular dynamics simulation study of carbon dioxide, methane, and their mixture in the presence of brine. J. Phys. Chem. B 2017, 121, 9688–9698. 10.1021/acs.jpcb.7b08118. 28972373

[ref55] Papavasileiou K. D.; Moultos O. A.; Economou I. G. Predictions of water/oil interfacial tension at elevated temperatures and pressures: A molecular dynamics simulation study with biomolecular force fields. Fluid Phase Equilib. 2018, 476, 30–38. 10.1016/j.fluid.2017.05.004.

[ref56] Aminian A.; ZareNezhad B. Molecular dynamics simulations study on the shear viscosity, density, and equilibrium interfacial tensions of CO_2_ + brines and brines + CO_2_ + n-decane systems. J. Phys. Chem. B 2021, 125, 2707–2718. 10.1021/acs.jpcb.0c10883. 33689346

[ref57] Blazquez S.; Zeron I.; Conde M.; Abascal J.; Vega C. Scaled charges at work: Salting out and interfacial tension of methane with electrolyte solutions from computer simulations. Fluid Phase Equilib. 2020, 513, 112548 10.1016/j.fluid.2020.112548.

[ref58] Kallikragas D. T.; Plugatyr A. Y.; Svishchev I. M. High temperature diffusion coefficients for O_2_, H_2_, and OH in water, and for pure water. J. Chem. Eng. Data 2014, 59, 1964–1969. 10.1021/je500096r.

[ref59] Zhao X.; Jin H. Investigation of hydrogen diffusion in supercritical water: A molecular dynamics simulation study. Int. J. Heat Mass Transfer 2019, 133, 718–728. 10.1016/j.ijheatmasstransfer.2018.12.164.

[ref60] Tsimpanogiannis I. N.; Maity S.; Celebi A. T.; Moultos O. A. Engineering Model for Predicting the Intradiffusion Coefficients of Hydrogen and Oxygen in Vapor, Liquid, and Supercritical Water based on Molecular Dynamics Simulations. J. Chem. Eng. Data 2021, 66, 3244 10.1021/acs.jced.1c00300.

[ref61] Zhao X.; Jin H. Correlation for self-diffusion coefficients of H_2_, CH_4_, CO, O_2_ and CO_2_ in supercritical water from molecular dynamics simulation. Appl. Therm. Eng. 2020, 171, 114941 10.1016/j.applthermaleng.2020.114941.

[ref62] Zhao X.; Jin H.; Chen Y.; Ge Z. Numerical study of H_2_, CH_4_, CO, O_2_ and CO_2_ diffusion in water near the critical point with molecular dynamics simulation. Comput. Math. with Appl. 2021, 81, 759–771. 10.1016/j.camwa.2019.11.012.

[ref63] Marx D.; Nielaba P. Path-integral Monte Carlo techniques for rotational motion in two dimensions: Quenched, annealed, and no-spin quantum-statistical averages. Phys. Rev. A 1992, 45, 8968 10.1103/PhysRevA.45.8968. 9907002

[ref64] Köster A.; Thol M.; Vrabec J. Molecular Models for the Hydrogen Age: Hydrogen, Nitrogen, Oxygen, Argon, and Water. J. Chem. Eng. Data 2018, 63, 305–320. 10.1021/acs.jced.7b00706.

[ref65] Buch V. Path integral simulations of mixed para-D_2_ and ortho-D–2 clusters: The orientational effects. J. Chem. Phys. 1994, 100, 7610–7629. 10.1063/1.466854.

[ref66] Hirschfelder J. O.; Curtiss C. F.; Bird R. B. Molecular theory of gases and liquids, 1st ed.; John Wiley: New York, 1964.

[ref67] Cracknell R. F. Molecular Simulation of hydrogen adsorption in graphitic nanofibres. Phys. Chem. Chem. Phys. 2001, 3, 2091–2097. 10.1039/b100144m.

[ref68] Alavi S.; Ripmeester J.; Klug D. Molecular-dynamics study of structure II hydrogen clathrates. J. Chem. Phys. 2005, 123, 024507 10.1063/1.1953577. 16050759

[ref69] Abascal J. L.; Vega C. A general purpose model for the condensed phases of water: TIP4P/2005. J. Chem. Phys. 2005, 123, 234505 10.1063/1.2121687. 16392929

[ref70] Garcia-Ratés M.; de Hemptinne J.-C.; Avalos J. B.; Nieto-Draghi C. Molecular Modeling of Diffusion Coefficient and Ionic Conductivity of CO_2_ in Aqueous Ionic Solutions. J. Phys. Chem. B 2012, 116, 2787–2800. 10.1021/jp2081758. 22292779

[ref71] Aimoli C. G.; Maginn E. J.; Abreu C. R. Transport properties of carbon dioxide and methane from molecular dynamics simulations. J. Chem. Phys. 2014, 141, 134101 10.1063/1.4896538. 25296778

[ref72] Zhong H.; Lai S.; Wang J.; Qiu W.; Ludemann H.-D.; Chen L. Molecular dynamics simulation of transport and structural properties of CO_2_ using different molecular models. J. Chem. Eng. Data 2015, 60, 2188–2196. 10.1021/je5009526.

[ref73] Jiang H.; Economou I. G.; Panagiotopoulos A. Z. Molecular modeling of thermodynamic and transport properties for CO_2_ and aqueous brines. Acc. Chem. Res. 2017, 50, 751–758. 10.1021/acs.accounts.6b00632. 28234455

[ref74] Lopez-Lazaro C.; Bachaud P.; Moretti I.; Ferrando N. Predicting the phase behavior of hydrogen in NaCl brines by Molecular Simulation for geological applications. BSGF-Earth Sci. Bulletin 2019, 190, 7 10.1051/bsgf/2019008.

[ref75] Liu Y.; Panagiotopoulos A. Z.; Debenedetti P. G. Monte Carlo simulations of high-pressure phase equilibria of CO_2_-H_2_O mixtures. J. Phys. Chem. B 2011, 115, 6629–6635. 10.1021/jp201520u. 21528884

[ref76] Liu Y.; Lafitte T.; Panagiotopoulos A. Z.; Debenedetti P. G. Simulations of vapor-liquid phase equilibrium and interfacial tension in the CO_2_-H_2_O-NaCl system. AIChE J. 2013, 59, 3514–3522. 10.1002/aic.14042.

[ref77] Habibi P.; Rahbari A.; Blazquez S.; Vega C.; Dey P.; Vlugt T. J. H.; Moultos O. A. A New Force Field for OH- for Computing Thermodynamic and Transport Properties of H_2_ and O_2_ in Aqueous NaOH and KOH Solutions. J. Phys. Chem. B 2022, 126, 9376–9387. 10.1021/acs.jpcb.2c06381. 36325986 PMC9677430

[ref78] Rahbari A.; Hens R.; Ramdin M.; Moultos O. A.; Dubbeldam D.; Vlugt T. J. H. Recent advances in the Continuous Fractional Component Monte Carlo methodology. Mol. Simul. 2021, 47, 804–823. 10.1080/08927022.2020.1828585.

[ref79] Shi W.; Maginn E. J. Continuous Fractional Component Monte Carlo: an adaptive biasing method for open system atomistic simulations. J. Chem. Theory Comput. 2007, 3, 1451–1463. 10.1021/ct7000039. 26633216

[ref80] Shi W.; Maginn E. J. Improvement in molecule exchange efficiency in Gibbs ensemble Monte Carlo: Development and implementation of the Continuous Fractional Component move. J. Comput. Chem. 2008, 29, 2520–2530. 10.1002/jcc.20977. 18478586

[ref81] Zeron I. M.; Abascal J. L. F.; Vega C. A force field of Li^+^, Na^+^, K^+^, Mg^2+^, Ca^+^, Cl^–^, and SO_4_^2–^ in aqueous solution based on the TIP4P/2005 water model and scaled charges for the ions. J. Chem. Phys. 2019, 151, 104501 10.1063/1.5121392. 31594349

[ref82] Blazquez S.; Conde M. M.; Vega C. Unpublished. 2023.

[ref83] Vega C.; Abascal J. L. Simulating water with rigid non-polarizable models: a general perspective. Phys. Chem. Chem. Phys. 2011, 13, 19663–19688. 10.1039/c1cp22168j. 21927736

[ref84] Tsimpanogiannis I. N.; Moultos O. A.; Franco L. F.; Spera M. B. M.; Erdös M.; Economou I. G. Self-diffusion coefficient of bulk and confined water: a critical review of classical Molecular Simulation studies. Mol. Simul. 2019, 45, 425–453. 10.1080/08927022.2018.1511903.

[ref85] González M. A.; Abascal J. L. The shear viscosity of rigid water models. J. Chem. Phys. 2010, 132, 096101 10.1063/1.3330544. 20210414

[ref86] Moultos O. A.; Tsimpanogiannis I. N.; Panagiotopoulos A. Z.; Economou I. G. Atomistic molecular dynamics simulations of CO_2_ diffusivity in H_2_O for a wide range of temperatures and pressures. J. Phys. Chem. B 2014, 118, 5532–5541. 10.1021/jp502380r. 24749622

[ref87] Sakamaki R.; Sum A. K.; Narumi T.; Ohmura R.; Yasuoka K. Thermodynamic properties of methane/water interface predicted by molecular dynamics simulations. J. Chem. Phys. 2011, 134, 144702 10.1063/1.3579480. 21495767

[ref88] Vega C.; de Miguel E. Surface tension of the most popular models of water by using the test-area simulation method. J. Chem. Phys. 2007, 126, 154707 10.1063/1.2715577. 17461659

[ref89] Döpke M. F.; Moultos O. A.; Hartkamp R. On the transferability of ion parameters to the TIP4P/2005 water model using molecular dynamics simulations. J. Chem. Phys. 2020, 152, 024501 10.1063/1.5124448. 31941316

[ref90] Deeg K. S.; Gutiérrez-Sevillano J. J.; Bueno-Pérez R.; Parra J. B.; Ania C. O.; Doblaré M.; Calero S. Insights on the Molecular Mechanisms of Hydrogen Adsorption in Zeolites. J. Phys. Chem. C 2013, 117, 14374–14380. 10.1021/jp4037233.

[ref91] Sesé L. M. Study of the Feynman-Hibbs effective potential against the path-integral formalism for Monte Carlo simulations of quantum many-body Lennard-Jones systems. Mol. Phys. 1994, 81, 1297–1312. 10.1080/00268979400100891.

[ref92] Sesé L. M. Feynman-Hibbs potentials and path integrals for quantum Lennard-Jones systems: Theory and Monte Carlo simulations. Mol. Phys. 1995, 85, 931–947. 10.1080/00268979500101571.

[ref93] Rahbari A.; Brenkman J.; Hens R.; Ramdin M.; van den Broeke L. J. P.; Schoon R.; Henkes R.; Moultos O. A.; Vlugt T. J. H. Solubility of water in hydrogen at high pressures: A Molecular Simulation study. J. Chem. Eng. Data 2019, 64, 4103–4115. 10.1021/acs.jced.9b00513.

[ref94] Plimpton S. Fast Parallel Algorithms for Short-Range Molecular Dynamics. J. Comput. Phys. 1995, 117, 1–19. 10.1006/jcph.1995.1039.

[ref95] Ryckaert J. P.; Ciccotti G.; Berendsen H. J. Numerical integration of the cartesian equations of motion of a system with constraints: molecular dynamics of n-alkanes. J. Comput. Phys. 1977, 23, 327–341. 10.1016/0021-9991(77)90098-5.

[ref96] Allen M. P.; Tildesley D. J. Computer simulation of liquids; Oxford University Press, 2017.

[ref97] Frenkel D.; Smit B. Understanding Molecular Simulation: from algorithms to applications, 2nd ed.; Elsevier: San Diego, 2002.

[ref98] Hockney R.; Eastwood J. Computer Simulation Using Particles, 1st ed.; CRC Press: New York, 1988.

[ref99] Plimpton S. LAMMPS Documentation (15 Sep 2022 version). https://docs.lammps.org/Manual.html (accessed: 26/10/2022).

[ref100] Martinez L.; Andrade R.; Birgin E. G.; Martínez J. M. PACKMOL: A package for building initial configurations for molecular dynamics simulations. J. Comput. Chem. 2009, 30, 2157–2164. 10.1002/jcc.21224. 19229944

[ref101] Isele-Holder R. E.; Mitchell W.; Ismail A. E. Development and application of a particle-particle particle-mesh Ewald method for dispersion interactions. J. Chem. Phys. 2012, 137, 174107 10.1063/1.4764089. 23145717

[ref102] Salehi H. S.; Moultos O. A.; Vlugt T. J. H. Interfacial Properties of Hydrophobic Deep Eutectic Solvents with Water. J. Phys. Chem. B 2021, 125, 12303–12314. 10.1021/acs.jpcb.1c07796. 34719232 PMC8591605

[ref103] Rowlinson J. S.; Widom B. Molecular theory of capillarity, 1st ed.; Courier Corporation: Oxford, 1982.

[ref104] Jamali S. H.; Wolff L.; Becker T. M.; de Groen M.; Ramdin M.; Hartkamp R.; Bardow A.; Vlugt T. J. H.; Moultos O. A. OCTP: ATool for On-the-Fly Calculation of Transport Properties of Fluids with the Order- n Algorithm in LAMMPS. J. Chem. Inf. Model. 2019, 59, 1290–1294. 10.1021/acs.jcim.8b00939. 30742429

[ref105] Dubbeldam D.; Ford D. C.; Ellis D. E.; Snurr R. Q. A new perspective on the order-n algorithm for computing correlation functions. Mol. Simul. 2009, 35, 1084–1097. 10.1080/08927020902818039.

[ref106] Yeh I.-C.; Hummer G. System-size dependence of diffusion coefficients and viscosities from molecular dynamics simulations with periodic boundary conditions. J. Phys. Chem. B 2004, 108, 15873–15879. 10.1021/jp0477147.

[ref107] Celebi A. T.; Jamali S. H.; Bardow A.; Vlugt T. J. H.; Moultos O. A. Finite-size effects of diffusion coefficients computed from molecular dynamics: a review of what we have learned so far. Mol. Simul. 2021, 47, 831–845. 10.1080/08927022.2020.1810685.

[ref108] Jamali S. H.; Bardow A.; Vlugt T. J. H.; Moultos O. A. Generalized form for finite-size corrections in mutual diffusion coefficients of multicomponent mixtures obtained from equilibrium molecular dynamics simulation. J. Chem. Theory Comput. 2020, 16, 3799–3806. 10.1021/acs.jctc.0c00268. 32338889 PMC7288667

[ref109] Moultos O. A.; Zhang Y.; Tsimpanogiannis I. N.; Economou I. G.; Maginn E. J. System-size corrections for self-diffusion coefficients calculated from molecular dynamics simulations: The case of CO_2_, n-alkanes, and poly (ethylene glycol) dimethyl ethers. J. Chem. Phys. 2016, 145, 074109 10.1063/1.4960776. 27544089

[ref110] Jamali S. H.; Hartkamp R.; Bardas C.; Sohl J.; Vlugt T. J. H.; Moultos O. A. Shear viscosity computed from the finite-size effects of self-diffusivity in equilibrium molecular dynamics. J. Chem. Theory Comput. 2018, 14, 5959–5968. 10.1021/acs.jctc.8b00625. 30296092 PMC6236468

[ref111] Jamali S. H.; Wolff L.; Becker T. M.; Bardow A.; Vlugt T. J. H.; Moultos O. A. Finite-size effects of binary mutual diffusion coefficients from molecular dynamics. J. Chem. Theory Comput. 2018, 14, 2667–2677. 10.1021/acs.jctc.8b00170. 29664633 PMC5943679

[ref112] Hens R.; Rahbari A.; Caro-Ortiz S.; Dawass N.; Erdos M.; Poursaeidesfahani A.; Salehi H. S.; Celebi A. T.; Ramdin M.; Moultos O. A.; Dubbeldam D.; Vlugt T. J. H. Brick-CFCMC: Open Source Software for Monte Carlo Simulations of Phase and Reaction Equilibria Using the Continuous Fractional Component Method. J. Chem. Inf. Model. 2020, 60, 2678–2682. 10.1021/acs.jcim.0c00334. 32275829 PMC7312392

[ref113] Polat H. M.; Salehi H. S.; Hens R.; Wasik D. O.; Rahbari A.; de Meyer F.; Houriez C.; Coquelet C.; Calero S.; Dubbeldam D.; Moultos O. A.; Vlugt T. J. H. New Features of the Open Source Monte Carlo Software Brick-CFCMC: Thermodynamic Integration and Hybrid Trial Moves. J. Chem. Inf. Model. 2021, 61, 3752–3757. 10.1021/acs.jcim.1c00652. 34383501 PMC8385706

[ref114] Poursaeidesfahani A.; Hens R.; Rahbari A.; Ramdin M.; Dubbeldam D.; Vlugt T. J. H. Efficient application of Continuous Fractional Component Monte Carlo in the reaction ensemble. J. Chem. Theory Comput. 2017, 13, 4452–4466. 10.1021/acs.jctc.7b00092. 28737933 PMC5597954

[ref115] Rahbari A.; Hens R.; Dubbeldam D.; Vlugt T. J. H. Improving the accuracy of computing chemical potentials in CFCMC simulations. Mol. Phys. 2019, 117, 3493–3508. 10.1080/00268976.2019.1631497.

[ref116] Rahbari A.; Hens R.; Jamali S.; Ramdin M.; Dubbeldam D.; Vlugt T. J. H. Effect of truncating electrostatic interactions on predicting thermodynamic properties of water-methanol systems. Mol. Simul. 2019, 45, 336–350. 10.1080/08927022.2018.1547824.

[ref117] Wang F.; Landau D. P. Efficient multiple-range random walk algorithm to calculate the density of states. Phys. Rev. Lett. 2001, 86, 2050 10.1103/PhysRevLett.86.2050. 11289852

[ref118] Wang F.; Landau D. P. Determining the density of states for classical statistical models: A random walk algorithm to produce a flat histogram. Phys. Rev. E 2001, 64, 056101 10.1103/PhysRevE.64.056101. 11736008

[ref119] Salehi H. S.; Hens R.; Moultos O. A.; Vlugt T. J. H. Computation of gas solubilities in choline chloride urea and choline chloride ethylene glycol deep eutectic solvents using Monte Carlo simulations. J. Mol. Liq. 2020, 316, 113729 10.1016/j.molliq.2020.113729.

[ref120] Widom B. Potential-distribution theory and the statistical mechanics of fluids. J. Phys. Chem. 1982, 86, 869–872. 10.1021/j100395a005.

[ref121] Widom B. Some Topics in the Theory of Fluids. J. Chem. Phys. 1963, 39, 2808–2812. 10.1063/1.1734110.

[ref122] Rahbari A.; Hens R.; Nikolaidis I. K.; Poursaeidesfahani A.; Ramdin M.; Economou I. G.; Moultos O. A.; Dubbeldam D.; Vlugt T. J. H. Computation of partial molar properties using continuous fractional component Monte Carlo. Mol. Phys. 2018, 116, 3331–3344. 10.1080/00268976.2018.1451663.

[ref123] Georgiadis A.; Maitland G.; Trusler J. M.; Bismarck A. Interfacial tension measurements of the (H_2_O+ CO_2_) system at elevated pressures and temperatures. J. Chem. Eng. Data 2010, 55, 4168–4175. 10.1021/je100198g.

[ref124] Bachu S.; Bennion D. B. Interfacial tension between CO_2_, freshwater, and brine in the range of pressure from (2 to 27) MPa, temperature from (20 to 125) °C, and water salinity from (0 to 334 000) mg· L^–1^. J. Chem. Eng. Data 2009, 54, 765–775. 10.1021/je800529x.

[ref125] Hebach A.; Oberhof A.; Dahmen N.; Kögel A.; Ederer H.; Dinjus E. Interfacial tension at elevated pressures measurements and correlations in the water+ carbon dioxide system. J. Chem. Eng. Data 2002, 47, 1540–1546. 10.1021/je025569p.

[ref126] Ren Q.-Y.; Chen G.-J.; Yan W.; Guo T.-M. Interfacial tension of (CO_2_+ CH_4_)+ water from 298 to 373 K and pressures up to 30 MPa. J. Chem. Eng. Data 2000, 45, 610–612. 10.1021/je990301s.

[ref127] Naeiji P.; Woo T. K.; Alavi S.; Ohmura R. Molecular dynamics simulations of interfacial properties of the CO_2_-water and CO_2_-CH_4_-water systems. J. Chem. Phys. 2020, 153, 044701 10.1063/5.0008114. 32752701

[ref128] Li X.; Boek E.; Maitland G. C.; Trusler J. P. M. Interfacial Tension of (Brines+ CO_2_):(0.864 NaCl+ 0.136 KCl) at Temperatures between (298 and 448) K, Pressures between (2 and 50) MPa, and Total Molalities of (1 to 5) mol· kg^–1^. J. Chem. Eng. Data 2012, 57, 1078–1088. 10.1021/je201062r.

[ref129] Aggelopoulos C. A.; Robin M.; Vizika O. Interfacial tension between CO_2_ and brine (NaCl+ CaCl_2_) at elevated pressures and temperatures: The additive effect of different salts. Adv. Water Resour. 2011, 34, 505–511. 10.1016/j.advwatres.2011.01.007.

[ref130] Jerauld G. R.; Kazemi A. An improved simple correlation for accurate estimation of CO_2_-Brine interfacial tension at reservoir conditions. J. Pet. Sci. Eng. 2022, 208, 109537 10.1016/j.petrol.2021.109537.

[ref131] Manciu M.; Ruckenstein E. Specific ion effects via ion hydration: I. Surface tension. Adv. Colloid Interface Sci. 2003, 105, 63–101. 10.1016/S0001-8686(03)00018-6. 12969642

[ref132] Marcus Y. Effect of ions on the structure of water: Structure making and breaking. Chem. Rev. 2009, 109, 1346–1370. 10.1021/cr8003828. 19236019

[ref133] Pegram L. M.; Record M. T. Hofmeister salt effects on surface tension arise from partitioning of anions and cations between bulk water and the air-water interface. J. Phys. Chem. B 2007, 111, 5411–5417. 10.1021/jp070245z. 17432897

[ref134] Levin Y.; dos Santos A. P.; Diehl A. Ions at the air-water interface: An end to a hundred-year-old mystery?. Phys. Rev. Lett. 2009, 103, 257802 10.1103/PhysRevLett.103.257802. 20366288

[ref135] Weisenberger S.; Schumpe A. Estimation of gas solubilities in salt solutions at temperatures from 273 to 363 K. AIChE J. 1996, 42, 298–300. 10.1002/aic.690420130.

[ref136] Laliberté M. Model for calculating the viscosity of aqueous solutions. J. Chem. Eng. Data 2007, 52, 321–335. 10.1021/je0604075.

[ref137] Laliberté M.; Cooper W. E. Model for calculating the density of aqueous electrolyte solutinos. J. Chem. Eng. Data 2004, 49, 1141–1151. 10.1021/je0498659.

[ref138] Tsimpanogiannis I. N.; Moultos O. A. Is Stokes-Einstein relation valid for the description of intra-diffusivity of hydrogen and oxygen in liquid water?. Fluid Phase Equilib. 2022, 563, 113568 10.1016/j.fluid.2022.113568.

[ref139] Cussler E. L. Diffusion Coefficients and Diffusion of Interacting Species, 2nd ed.; Cambridge University Press: Cambridge, 2009.

[ref140] Taylor R.; Krishna R. Multicomponent mass transfer, 1st ed.; John Wiley & Sons, 1993; Vol. 2.

[ref141] Krishna R.; Wesselingh J. The Maxwell-Stefan approach to mass transfer. Chem. Eng. Sci. 1997, 52, 861–911. 10.1016/S0009-2509(96)00458-7.

[ref142] Wiebe R.; Gaddy V. L. The Solubility of Hydrogen in Water at 0, 50, 75 and 100° from 25 to 1000 atm. J. Am. Chem. Soc. 1934, 56, 76–79. 10.1021/ja01316a022.

[ref143] Wiebe R.; Gaddy V.; Heins C. Solubility of Hydrogen in Water at 25° C. from 25 to 1000 atm. Ind. Eng. Chem. 1932, 24, 823–825. 10.1021/ie50271a024.

[ref144] Kling G.; Maurer G. The solubility of hydrogen in water and in 2-aminoethanol at temperatures between 323 and 423 K and pressures up to 16 MPa. J. Chem. Thermodyn. 1991, 23, 531–541. 10.1016/S0021-9614(05)80095-3.

[ref145] Choudhary V. R.; Parande M. G.; Brahme P. H. Simple apparatus for measuring solubility of gases at high pressures. Ind. Eng. Chem. Fundam. 1982, 21, 472–474. 10.1021/i100008a027.

[ref146] Chandrasekhar J.; Spellmeyer D. C.; Jorgensen W. L. Energy component analysis for dilute aqueous solutions of lithium (1+), sodium (1+), fluoride (1-), and chloride (1-) ions. J. Am. Chem. Soc. 1984, 106, 903–910. 10.1021/ja00316a012.

[ref147] Darkrim F.; Vermesse J.; Malbrunot P.; Levesque D. Monte Carlo simulations of nitrogen and hydrogen physisorption at high pressures and room temperature. Comparison with experiments. J. Chem. Phys. 1999, 110, 4020–4027. 10.1063/1.478283.

[ref148] Zhu Z.; Cao Y.; Zheng Z.; Chen D. An Accurate Model for Estimating H_2_ Solubility in Pure Water and Aqueous NaCl Solutions. Energies 2022, 15, 5021 10.3390/en15145021.

