# Supplementary material for: Interfacial Tensions, Solubilities, and Transport Properties of the H2/H2O/NaCl System: A Molecular Simulation Study
Source: J Chem Eng Data. 2023 Jan 11;69(2):307–19. doi: 10.1021/acs.jced.2c00707 (PMC10859954; doi:10.1021/acs.jced.2c00707)
Supplement: Supplementary file 1 — je2c00707_si_001.pdf [file je2c00707_si_001.pdf]

Supporting Information: Interfacial tensions, solubilities, and transport properties of the H_2/H_2O/NaCl system: A molecular simulation study

W. A. van Rooijen,[†,§] P. Habibi,[‡,¶,§] K. Xu,[¶] P. Dey,[¶] T. J. H. Vlugt,[‡] H. Hajibeygi,[†] and O. A. Moultos[*,‡]

†*Reservoir Engineering, Geoscience and Engineering Department, Faculty of Civil Engineering and Geosciences, Delft University of Technology, Stevinweg 1, 2628CN, Delft, The Netherlands*

‡*Engineering Thermodynamics, Process and Energy Department, Faculty of Mechanical, Maritime and Materials Engineering, Delft University of Technology, Leeghwaterstraat 39, 2628CB, Delft, The Netherlands*

¶*Department of Materials Science and Engineering, Faculty of Mechanical, Maritime and Materials Engineering, Delft University of Technology, Mekelweg 2, 2628CD, Delft, The Netherlands*

§*These authors contributed equally*

E-mail: o.moultos@tudelft.nl

Solubilities of H_2 in pure water (Table S1); Force field parameters (Tables S2 - S5); Additional info MD and CFCMC simulations (Tables S6 - S8); Raw data MD simulations for Interfacial Tensions (Table S9); Density and viscosity data (Table S10); Raw data MD simulations for self-diffusivities (Tables S11 - S12); Raw data CFCMC simulations (Table S13); Solubilities of H_2 in pure water (Figure S1); Density profile of Na^+ and Cl^- of a MD simulation to calculate interfacial tensions (Figure S2); Solubilities of H_2 in aqueous NaCl solution at H_2 partial pressures of 1-100 bar (Figure S3); Densities of NaCl solutions (Figure S4); Viscosities of NaCl solutions (Figure S5).

Table S1: Computed solubilities of H_2 at a H_2 partial pressure of 1 bar in pure water using the TIP4P/2005[1] H_2O force field in combination with the Marx[2] H_2 force field and the Vrabec[3] force field as a function of temperature T. T is in unit of K, x_{H_2} and $\sigma_{x_{H_2}}$ are reported with multiplication factors of 10^{-5}. $\sigma_{x_{H_2}}$ is the uncertainty of x_{H_2}. The last column lists the calculated solubilities using the experimental correlation provided by Torín-Ollarves and Trusler[4]

	Marx[2]		Vrabec[3]		Experimental[4]
T	x_{H_2}	$\sigma_{x_{H_2}}$	x_{H_2}	$\sigma_{x_{H_2}}$	x_{H_2}
298	1.30	0.03	0.66	0.01	1.39
323	1.19	0.02	0.68	0.02	1.29
333	-	-	0.69	0.01	1.29
343	1.20	0.02	0.72	0.03	1.32
353	-	-	0.75	0.01	1.36
363	1.28	0.02	-	-	1.42

Table S2: Parameters for the TIP4P/2005[1] water force field. σ and ϵ are the Lennard-Jones parameters, q is the atomic partial charge, and l is the bond length. σ and l are in units of Å, ϵ is in units of kJ/mol, and q is in units of the elementary charge e. In the TIP4P/2005[1] force field, the charge on O is on a massless site M.

$\mathrm{H}-\widehat{\mathrm{O}}-\mathrm{H}\,(^{o})$	104.52
$l_{\mathrm{O-H}}$	0.9572
$l_{\mathrm{O-M}}$	0.1546
σ_{OO}	3.1589
σ_{HH}	0
ϵ_{OO}	0.774908
ϵ_{HH}	0
q_{O}	0
q_{M}	-1.1128
q_{H}	0.5564

Table S3: Parameters for the single-site Vrabec[3] Hydrogen force field. σ and ϵ are the Lennard-Jones parameters. σ is in units of Å and ϵ is in units of kJ/mol.

σ_{HH}	3.0366
ϵ_{HH}	0.214846

Table S4: Parameters for the three-site Marx[2] Hydrogen force field. σ and ϵ are the Lennard-Jones parameters, q is the atomic partial charge, dummy site L is the geometric center of mass, and l is the bond length. σ and l are in units of Å, ϵ is in units of kJ/mol, and q is in units of the elementary charge e.

σ_{LL}	2.958
ϵ_{LL}	0.305141
q_{H}	0.468
q_{L}	-0.936
$l_{\mathrm{H-H}}$	0.74

Table S5: Parameters for the Madrid-Transport[5,6] and Madrid-2019[7] force fields. σ and ϵ are the Lennard-Jones parameters and q is the atomic partial charge. σ is units of Å, ϵ is in units of kJ/mol, and q is in units of the elementary charge e.

	Madrid-Transport	Madrid-2019
$\sigma_{\mathrm{Na^+Na^+}}$	2.21737	2.21737
$\sigma_{\mathrm{Na^+Cl^-}}$	2.58012	3.00512
$\sigma_{\mathrm{Cl^-Cl^-}}$	4.69906	4.69906
$\sigma_{\mathrm{Na^+O_w}}$	2.38725	2.60838
$\sigma_{\mathrm{Cl^-O_w}}$	4.07631	4.23867
$\epsilon_{\mathrm{Na^+Na^+}}$	1.472356	1.472356
$\epsilon_{\mathrm{Na^+Cl^-}}$	1.438894	1.438894
$\epsilon_{\mathrm{Cl^-Cl^-}}$	0.076923	0.076923
$\epsilon_{\mathrm{Na^+O_w}}$	0.793388	0.793388
$\epsilon_{\mathrm{Cl^-O_w}}$	0.061983	0.061983
$q_{\mathrm{Na^+}}$	0.75	0.85
$q_{\mathrm{Cl^-}}$	-0.75	-0.85

Table S6: The numbers of H_2, H_2O molecules, and Na^+ and Cl^- ions N_{H_2}, N_{H_2O}, N_{Na^+/Cl^-}, used in the MD simulations to compute interfacial tensions for a wide range of pressures P, temperatures T, and NaCl molalities m_{NaCl}. The different columns represent different NaCl molalities. The last column reports the average simulation cell sizes L_x, L_y and L_z for a molality of 1.01 mol NaCl/kg H_2O. T is in units of K, P is in units of bar, m_{NaCl} is in units of mol NaCl/kg H_2O, and L is in Å.

		N_{H_2}	N_{H_2O}	N_{Na^+/Cl^-}	N_{H_2}	N_{H_2O}	N_{Na^+/Cl^-}	N_{H_2}	N_{H_2O}	N_{Na^+/Cl^-}	N_{H_2}	N_{H_2O}	N_{Na^+/Cl^-}	$L_x \times L_y \times L_z$
T	m_{NaCl} P	0.00			1.01			3.00			5.00			1.01
298	1	64	2088	0	64	2088	38	64	2090	113	64	2090	188	39 × 39 × 101
	10	320	2088	0	320	2088	38	320	2090	113	320	2090	188	39 × 39 × 430
	100	640	2088	0	640	2088	38	640	2090	113	640	2090	188	39 × 39 × 203
	200	640	2088	0	640	2088	38	640	2090	113	640	2090	188	39 × 39 × 139
	400	640	2088	0	640	2088	38	640	2090	113	640	2090	188	39 × 39 × 96
	600	640	2088	0	640	2088	38	640	2090	113	640	2090	188	39 × 39 × 83
323	1	64	2088	0	64	2088	38	64	2090	113	64	2090	188	39 × 39 × 118
	10	320	2088	0	320	2088	38	320	2090	113	320	2090	188	39 × 39 × 468
	100	640	2088	0	640	2088	38	640	2090	113	640	2090	188	39 × 39 × 216
	138	-	-	-	640	2088	38	-	-	-	-	-	-	39 × 39 × 179
	400	640	2088	0	640	2088	38	640	2090	113	640	2090	188	39 × 39 × 147
	276	-	-	-	640	2088	38	-	-	-	-	-	-	39 × 39 × 124
	400	640	2088	0	640	2088	38	640	2090	113	640	2090	188	39 × 39 × 100
	600	640	2088	0	640	2088	38	640	2090	113	640	2090	188	39 × 39 × 86
343	1	64	2088	0	64	2088	38	64	2090	113	64	2090	188	39 × 39 × 121
	10	320	2088	0	320	2088	38	320	2090	113	320	2090	188	39 × 39 × 508
	100	640	2088	0	640	2088	38	640	2090	113	640	2090	188	39 × 39 × 228
	200	640	2088	0	640	2088	38	640	2090	113	640	2090	188	39 × 39 × 152
	400	640	2088	0	640	2088	38	640	2090	113	640	2090	188	39 × 39 × 103
	600	640	2088	0	640	2088	38	640	2090	113	640	2090	188	39 × 39 × 88
373	1	64	2088	0	64	2088	38	64	2090	113	64	2090	188	39 × 39 × 114
	10	320	2088	0	320	2088	38	320	2090	113	320	2090	188	39 × 39 × 629
	100	640	2088	0	640	2088	38	640	2090	113	640	2090	188	39 × 39 × 246
	138	-	-	-	640	2088	38	-	-	-	-	-	-	39 × 39 × 200
	200	640	2088	0	640	2088	38	640	2090	113	640	2090	188	39 × 39 × 162
	276	-	-	-	640	2088	38	-	-	-	-	-	-	39 × 39 × 136
	400	640	2088	0	640	2088	38	640	2090	113	640	2090	188	39 × 39 × 112
	600	640	2088	0	640	2088	38	640	2090	113	640	2090	188	39 × 39 × 92
423	100	640	2088	0	640	2088	38	640	2090	113	640	2090	188	39 × 39 × 281
	200	640	2088	0	640	2088	38	640	2090	113	640	2090	188	39 × 39 × 178
	400	640	2088	0	640	2088	38	640	2090	113	640	2090	188	39 × 39 × 121
	600	640	2088	0	640	2088	38	640	2090	113	640	2090	188	39 × 39 × 99
523	200	640	2088	0	640	2088	38	640	2090	113	640	2090	188	39 × 39 × 228
	400	640	2088	0	640	2088	38	640	2090	113	640	2090	188	39 × 39 × 145
	600	640	2088	0	640	2088	38	640	2090	113	640	2090	188	39 × 39 × 116

Table S7: The numbers of molecules or ions N used in the MD simulations to compute self-diffusivities. m_{NaCl} is in units of mol NaCl/kg water. For each molality, the same numbers of molecules and ions are used for all temperatures and pressures

m_{NaCl}	N_{H_2}	N_{H_2O}	N_{Na^+}	N_{Cl^-}
0	2	700	0	0
1.03	2	700	13	13
3.01	2	700	38	38
5.00	2	700	63	63
6.03	2	700	76	76

Table S8: The numbers of molecules or ions N used in the CFCMC simulations. m_{NaCl} is in units of mol NaCl/kg water. For each molality, the same numbers of molecules and ions are used for all temperatures and pressures. In every simulation, a single fractional molecule of H_2 used.

m_{NaCl}	N_{H_2O}	N_{Na^+}	N_{Cl^-}
0	300	0	0
0.93	300	5	5
2.96	300	16	16
5.00	300	27	27
5.93	300	32	32

Table S9: The interfacial tensions γ of H_2 in contact with aqueous NaCl solutions as computed using the NaCl Madrid-2019[7] force field, the TIP4P/2005[1] water force field, and the Vrabec[3] hydrogen force field. T is in units of K, P is in units of bar, m_{NaCl} is in units of mol NaCl/kg H_2O, and γ is in units of mN/m. σ_γ is the uncertainty of γ. Conditions at which simulations are not performed are denoted by -. Uncertainties in pressure and temperature are zero as these are imposed in the simulations (NPT ensemble).

		γ	σ_γ	γ	σ_γ	γ	σ_γ	γ	σ_γ
	m_{NaCl}	0.00		1.01		3.00		5.00	
T	P								
298	1	64.9	0.7	67	1	69	1	73	1
	10	66	1	68	2	70.3	0.8	72.8	0.4
	100	65	1	68	1	69	2	73	1
	200	65.1	0.7	66	2	68.4	0.8	71.3	0.9
	400	64.0	0.8	66.6	0.8	69	1	70	1
	600	65	1	66	1	68.4	0.6	72.0	0.7
323	1	62.3	0.9	63.5	0.7	66.5	0.8	70	1
	10	61.9	0.1	64.4	0.8	66.6	0.9	69.9	0.7
	100	61.5	0.9	63	1	66.3	0.6	68	2
	138	-	-	63.7	0.6	-	-	-	-
	400	61.6	0.6	62.9	0.8	65.0	0.8	68	1
	276	-	-	63	1	-	-	-	-
	400	61.0	0.3	63	1	65.4	0.8	69	2
	600	61.0	0.5	63	1	65.0	0.9	70	1
343	1	58.6	0.6	60.0	0.8	63.2	0.3	66.1	0.5
	10	58.9	0.5	60.9	0.9	64.1	0.4	64.7	0.9
	100	58.8	0.4	60.7	0.8	62.8	0.8	66	1
	200	58	1	59.5	0.5	63	1	64.1	0.9
	400	58	1	60.4	0.8	63.1	0.5	65	1
	600	59.3	0.6	59.9	0.9	63	1	67.6	0.7
373	1	53.2	0.5	55.6	0.6	58.4	0.8	61	1
	10	54.3	0.2	55	1	57.8	0.4	61.0	0.9
	100	54.3	0.4	55.8	0.9	57.4	0.6	61.6	0.4
	138	-	-	56	1	-	-	-	-
	200	53.7	0.4	55.1	0.4	58.6	0.4	61	1
	276	-	-	56	1	-	-	-	-
	400	53.1	0.2	55.2	0.4	58.5	0.4	60.6	0.5
	600	54.1	0.6	55.4	0.4	58.1	0.6	61.2	0.4
423	100	44.0	0.8	46.3	0.5	49.6	0.9	52.1	0.7
	200	45.2	0.9	47.3	0.4	50.1	0.5	53.7	0.6
	400	45.2	0.5	47.6	0.6	50.3	0.7	53	1
	600	45.3	0.5	47.8	0.9	50.6	0.5	54	1
523	200	25	1	27.5	0.9	31.6	0.8	34.5	0.7
	400	25.4	0.4	27.2	0.4	32.0	0.1	35.6	0.7
	600	26.0	0.6	29.5	0.5	32.7	0.8	36	1

Table S10: The densities ρ and viscosities η of aqueous NaCl solutions as computed using the NaCl Madrid-2019[7] and Madrid-Transport[5,6] force fields, compared with a fit to experimental data for densities[8] and viscosities.[9] The TIP4P/2005[1] H_2O force field and the Vrabec[3] H_2 force field were used. T is in units of K, P is in units of bar, m_{NaCl} is in units of mol NaCl/kg H_2O, ρ is in units of kg/m^3 and η is in units of mPa·s. σ_x is the uncertainty of quantity x.

		Madrid-2019[7]				Madrid-Transport[5,6]				Experimental[8,9]	
T	m_{NaCl}	ρ	σ_ρ	η	σ_η	ρ	σ_ρ	η	σ_η	ρ	η
298	0.00	994	1	0.888	0.008	993.9	0.5	0.83	0.05	997.0	0.890
	0.93	1034.0	0.4	0.96	0.03	1032.4	0.4	0.969	0.003	1036.2	0.927
	2.96	1103.0	0.5	1.5	0.1	1100.8	0.4	1.22	0.05	1106.1	1.198
	5.00	1163.5	0.5	2.2	0.2	1162.1	0.6	1.5	0.1	1166.3	1.527
	5.93	1191.6	0.5	2.6	0.4	1192.3	0.4	1.8	0.2	1193.3	1.754
323	0.00	973.0	0.7	0.393	0.001	973.4	0.3	0.40	0.02	977.8	0.404
	0.93	1010.6	0.3	0.47	0.02	1010.2	0.1	0.50	0.05	1015.1	0.452
	2.93	1075.3	0.3	0.64	0.02	1076.8	0.3	0.57	0.06	1082.5	0.566
	5.00	1132.4	0.3	0.84	0.03	1136.8	0.3	0.72	0.04	1141.5	0.708
	5.93	1162.6	0.3	0.95	0.07	1165.9	0.4	0.78	0.05	1168.3	0.794

Table S11: Results of MD simulations to compute self-diffusivities of H_2 in aqueous NaCl solutions as computed using the NaCl Madrid-Transport[5,6] force field, the TIP4P/2005[1] H_2O force field, and the Vrabec[3] H_2 force field. The densities ρ, viscosities η, and H_2 finite-size corrected[10] self-diffusion coefficients $D_{\mathrm{H_2}}$ for a wide range of temperatures T, pressures P, NaCl molalities m_{NaCl}, and corresponding molarities M_{NaCl}. T is in units of K, P is in units of bar, m_{NaCl} is in units of mol NaCl/kg water, M_{NaCl} is in units of mol NaCl/L solution, ρ is in units of kg/m^3, η is in units of mPa·s, and D is in units of 10^{-9} m^2/s. σ_x is the uncertainty of quantity x. Uncertainties in pressure and temperature are zero as these are imposed in the simulations (NPT ensemble).

T	P	m_{NaCl}	M_{NaCl}	σ_M	ρ	σ_ρ	η	σ_η	$D_{\mathrm{H_2}}$	$\sigma_{D_{\mathrm{H_2}}}$
298	1	0	0	0	993.9	0.5	0.82	0.05	4.4	0.6
298	1	1.03	1.0030	0.0004	1032.4	0.4	0.969	0.003	3.7	0.6
298	1	3.01	2.820	0.001	1100.8	0.4	1.22	0.05	3.2	0.1
298	1	5.00	4.492	0.002	1162.1	0.6	1.51	0.13	2.8	0.2
298	1	6.03	5.312	0.002	1192.3	0.4	1.81	0.16	2.5	0.1
298	10	0	0	0	994.6	0.4	0.85	0.02	4.6	0.2
298	10	1.03	1.0040	0.0002	1032.7	0.2	0.96	0.03	3.9	0.6
298	10	3.01	2.820	0.001	1100.8	0.5	1.24	0.06	3.0	0.3
298	10	5.00	4.492	0.002	1162.1	0.4	1.6	0.2	2.7	0.2

Table S11 continued from previous page

T	P	m_{NaCl}	M_{NaCl}	σ_m	ρ	σ_ρ	η	σ_η	$D_{\mathrm{H_2}}$	$\sigma_{D_{\mathrm{H_2}}}$
298	10	6.03	5.313	0.002	1192.4	0.6	1.8	0.1	2.6	0.2
298	100	0	0	0	998.0	0.4	0.83	0.03	4.3	0.1
298	100	1.03	1.0080	0.0003	1036.9	0.3	1.0	0.1	3.9	0.4
298	100	3.01	2.8290	0.0004	1104.6	0.2	1.24	0.05	3.3	0.1
298	100	5.00	4.508	0.002	1166.0	0.5	1.6	0.1	2.9	0.2
298	100	6.03	5.329	0.002	1196.0	0.4	1.9	0.2	2.5	0.2
298	400	0.00	0.0	0.0	1011.2	0.4	0.83	0.04	4.3	0.2
298	400	1.03	1.0210	0.0003	1050.0	0.3	1.0	0.1	3.7	0.2
298	400	3.01	2.862	0.001	1117.3	0.5	1.3	0.1	2.9	0.2
298	400	5.00	4.556	0.001	1178.5	0.2	1.61	0.09	2.5	0.2
298	400	6.03	5.384	0.002	1208.4	0.4	1.77	0.08	2.2	0.2
298	1000	0	0	0	1036.4	0.3	0.9	0.1	3.9	0.3
298	1000	1.03	1.0440	0.0002	1073.9	0.2	0.92	0.07	3.4	0.2
298	1000	3.01	2.923	0.001	1141.2	0.4	1.3	0.2	2.1	0.4
298	1000	5.00	4.647	0.002	1202.2	0.5	1.47	0.03	2.4	0.3
298	1000	6.03	5.488	0.002	1231.6	0.4	1.8	0.1	2.1	0.1
343	1	0	0	0	973.4	0.3	0.40	0.02	9.4	0.4
343	1	1.03	0.9819	0.0001	1010.2	0.1	0.50	0.05	7.8	1.4
343	1	3.01	2.758	0.001	1076.8	0.3	0.57	0.06	6.8	0.7
343	1	5.00	4.395	0.001	1136.8	0.3	0.72	0.04	6	1
343	1	6.03	5.195	0.002	1165.9	0.4	0.78	0.05	6.0	0.1
343	10	0	0	0	973.2	0.3	0.40	0.02	8.9	0.6
343	10	1.03	0.9826	0.0002	1010.9	0.2	0.45	0.02	8.8	0.2
343	10	3.01	2.760	0.001	1077.5	0.5	0.56	0.02	7.5	0.2
343	10	5.00	4.396	0.001	1137.1	0.2	0.73	0.03	6.2	0.3
343	10	6.03	5.197	0.002	1166.3	0.4	0.74	0.06	6.0	0.4
343	100	0	0	0	977.6	0.2	0.39	0.01	9.2	0.5
343	100	1.03	0.9867	0.0003	1015.1	0.3	0.46	0.03	8	1
343	100	3.01	2.770	0.001	1081.3	0.4	0.57	0.02	7.1	0.6
343	100	5.00	4.411	0.001	1140.9	0.2	0.71	0.07	6.3	0.4

Table S11 continued from previous page

T	P	m_{NaCl}	M_{NaCl}	σ_m	ρ	σ_ρ	η	σ_η	$D_{\mathrm{H_2}}$	$\sigma_{D_{\mathrm{H_2}}}$
343	100	6.03	5.213	0.001	1170.0	0.3	0.75	0.05	5.9	0.2
343	400	0	0	0	991.0	0.2	0.41	0.01	8.7	0.3
343	400	1.031	0.9995	0.0002	1028.3	0.2	0.46	0.03	8.2	0.2
343	400	3.01	2.803	0.001	1094.5	0.3	0.57	0.02	7.0	0.5
343	400	5.00	4.461	0.001	1154.0	0.2	0.71	0.06	5.4	0.5
343	400	6.03	5.271	0.002	1182.9	0.4	0.80	0.04	5.2	0.7
343	1000	0	0	0	1015.2	0.2	0.42	0.02	7.9	0.3
343	1000	1.031	1.0230	0.0001	1052.3	0.1	0.50	0.05	7.1	0.2
343	1000	3.01	2.864	0.001	1118.3	0.2	0.59	0.02	6.1	0.5
343	1000	5.00	4.555	0.002	1178.2	0.5	0.73	0.04	5.9	0.9
343	1000	6.03	5.377	0.001	1206.7	0.3	0.82	0.03	5.0	0.5
523	100	0	0	0	789.8	0.6	0.106	0.002	44	6
523	100	1.03	0.810	0.001	833.4	0.6	0.13	0.01	43.3	0.8
523	100	3.01	2.315	0.001	903.9	0.4	0.146	0.004	37	2
523	100	5.00	3.733	0.003	965.5	0.7	0.172	0.007	32	3
523	100	6.03	4.432	0.001	994.7	0.3	0.185	0.004	32.5	0.8
523	400	0	0	0	822.1	0.3	0.118	0.005	41	3
523	400	1.03	0.8383	0.0004	862.4	0.4	0.14	0.01	39	3
523	400	3.01	2.386	0.001	931.4	0.2	0.152	0.001	32	6
523	400	5.00	3.833	0.001	991.5	0.3	0.184	0.005	31	3
523	400	6.03	4.546	0.001	1020.3	0.3	0.197	0.005	31	2
523	1000	0	0	0	867.9	0.2	0.130	0.002	34	2
523	1000	1.03	0.8813	0.0002	906.7	0.2	0.141	0.005	30	5
523	1000	3.01	2.493	0.001	973.3	0.3	0.169	0.004	30	2
523	1000	5.00	3.990	0.001	1032.2	0.3	0.19	0.01	27.4	0.5
523	1000	6.03	4.725	0.001	1060.4	0.3	0.213	0.005	27	1
723	400	0	0	0	352	2	0.042	0.001	298	9
723	400	1.03	0.431	0.002	443	2	0.051	0.003	225	6
723	400	3.01	1.448	0.006	565	2	0.065	0.002	162	6
723	400	5.00	2.547	0.007	659	2	0.078	0.002	123	7

Table S11 continued from previous page

T	P	m_{NaCl}	M_{NaCl}	σ_m	ρ	σ_ρ	η	σ_η	$D_{\mathrm{H_2}}$	$\sigma_{D_{\mathrm{H_2}}}$
723	400	6.03	3.120	0.007	700	2	0.084	0.001	111	9
723	1000	0	0	0	609.2	0.5	0.072	0.002	112	3
723	1000	1.03	0.6409	0.0003	659.3	0.3	0.083	0.006	101	3
723	1000	3.01	1.888	0.003	737	1	0.092	0.003	88	1
723	1000	5.00	3.097	0.002	801.2	0.5	0.105	0.003	79	2
723	1000	6.03	3.706	0.003	831.7	0.6	0.111	0.002	74	2

Table S12: Results of MD simulations used to compute self-diffusivities of H_2 in aqueous NaCl solutions as computed using the NaCl Madrid-2019[7] force field, the TIP4P/2005[1] H_2O force field, and the Vrabec[3] H_2 force field. The densities ρ, viscosities η, and H_2 finite-size corrected[10] self-diffusion coefficients D_{H_2} for a wide range of temperatures T, pressures P, NaCl molalities m_{NaCl}, and corresponding molarities M_{NaCl}. T is in units of K, P is in units of bar, m_{NaCl} is in units of mol NaCl/kg H_2O, M_{NaCl} is in units of mol NaCl/L solution, ρ is in units of kg/m^3, η is in units of mPa·s and D is in units of 10^{-9} m^2/s. σ_x is the uncertainty of quantity x. Uncertainties in pressure and temperature are zero as these are imposed in the simulations (NPT ensemble).

T	P	m_{NaCl}	M_{NaCl}	σ_M	ρ	σ_ρ	η	σ_η	D_{H_2}	$\sigma_{D_{H_2}}$
298	1	1.03	1.0050	0.0003	1034.0	0.4	1.02	0.05	3.9	0.5
298	1	3.01	2.825	0.001	1103.0	0.5	1.43	0.09	2.8	0.3
298	1	5.00	4.498	0.002	1163.5	0.5	2.1	0.1	2.2	0.3
298	10	1.03	1.0050	0.0002	1034.3	0.2	1.1	0.1	3.6	0.3
298	10	3.01	2.825	0.001	1103.1	0.4	1.52	0.08	3.0	0.2
298	10	5.00	4.496	0.002	1163.1	0.4	2.0	0.1	2.1	0.2
298	100	1.03	1.0090	0.0002	1038.2	0.2	1.01	0.05	3.8	0.2
298	100	3.01	2.835	0.001	1107.0	0.5	1.43	0.03	2.9	0.3
298	100	5.00	4.509	0.002	1166.3	0.6	2.1	0.1	2.1	0.1
298	400	1.03	1.0210	0.0001	1050.2	0.1	0.9	0.1	3.4	0.3
298	400	3.01	2.862	0.001	1117.5	0.6	1.4	0.1	2.7	0.2
298	400	5.00	4.548	0.002	1176.5	0.6	2.12	0.07	2.1	0.2
298	1000	1.03	1.0430	0.0004	1073.2	0.5	1.02	0.03	2.9	0.4
298	1000	3.01	2.9160	0.0007	1138.3	0.3	1.6	0.2	2.1	0.6
298	1000	5.00	4.622	0.002	1195.6	0.5	2.2	0.1	1.7	0.1
343	1	1.03	0.9823	0.0003	1010.6	0.3	0.47	0.02	8.5	0.4
343	1	3.01	2.7540	0.0008	1075.3	0.3	0.64	0.02	6.9	0.4
343	1	5.00	4.378	0.001	1132.4	0.3	0.84	0.02	5.2	0.1
343	10	1.03	0.9827	0.0001	1011.0	0.1	0.48	0.02	8.5	0.5
343	10	3.01	2.7560	0.0007	1075.8	0.3	0.62	0.03	6.9	0.6
343	10	5.00	4.380	0.001	1133.1	0.4	0.9	0.1	5.3	0.3
343	100	1.03	0.9864	0.0003	1014.8	0.3	0.48	0.02	8.3	0.2
343	100	3.01	2.7650	0.0004	1079.5	0.1	0.65	0.05	5.9	0.8
343	100	5.00	4.394	0.001	1136.7	0.3	0.81	0.02	5.1	0.5

Table S12 continued from previous page

T	P	m_{NaCl}	M_{NaCl}	σ_m	ρ	σ_ρ	η	σ_η	$D_{\mathrm{H_2}}$	$\sigma_{D_{\mathrm{H_2}}}$
343	400	1.03	0.9989	0.0001	1027.7	0.1	0.51	0.06	7.5	0.6
343	400	3.01	2.7960	0.0003	1091.6	0.1	0.65	0.04	6.0	0.6
343	400	5.00	4.4370	0.0007	1147.6	0.2	0.9	0.1	5.0	0.5
343	1000	1.03	1.0210	0.0001	1050.6	0.1	0.51	0.01	7	1
343	1000	3.01	2.8500	0.0006	1112.8	0.2	0.67	0.06	6.3	0.8
343	1000	5.00	4.5160	0.0003	1168.1	0.1	0.86	0.04	4.3	0.3
523	100	1.03	0.8119	0.0003	835.3	0.3	0.123	0.003	42	2
523	100	3.01	2.320	0.001	905.6	0.6	0.155	0.003	32	3
523	100	5.00	3.731	0.001	965.2	0.3	0.20	0.01	31.9	0.7
523	400	1.03	0.8393	0.0002	863.5	0.2	0.134	0.001	39	1
523	400	3.01	2.3840	0.0005	930.8	0.2	0.164	0.002	32.9	0.6
523	400	5.00	3.817	0.001	987.5	0.3	0.198	0.005	30	1
523	1000	1.03	0.8808	0.0002	906.2	0.2	0.15	0.01	33.2	0.6
523	1000	3.01	2.4830	0.0006	969.4	0.2	0.183	0.005	29	1
523	1000	5.00	3.9570	0.0005	1023.5	0.1	0.217	0.007	24	2
723	400	1.03	0.451	0.001	464	1	0.0516	0.0002	204	5
723	400	3.01	1.541	0.006	602	2	0.070	0.001	128	9
723	400	5.00	2.692	0.001	696.3	0.3	0.091	0.002	99	6
723	1000	1.03	0.6459	0.0007	664.5	0.7	0.082	0.003	95	3
723	1000	3.01	1.910	0.001	745.6	0.5	0.098	0.001	82	3
723	1000	5.00	3.134	0.002	810.8	0.4	0.1163	0.0007	72.8	0.8

Table S13: Results of the CFCMC simulations using the NaCl Madrid-2019 force field,[7] TIP4P/2005[1] H_2O force field and Marx[2] H_2 force field. The fluid densities ρ, infinite dilution chemical potentials μ^{ex} of H_2, the solubilities of H_2 s_{H_2}, and mole fractions x_{H_2} are reported for a wide range of temperatures T, pressures P, and NaCl molalities m_{NaCl}, and corresponding molarities M_{NaCl}. The standard deviations σ are reported. T is in units of K, P is in units of bar, m_{NaCl} is in units of mol NaCl/kg water, M_{NaCl} is in units of mol NaCl/L solution, ρ is in units of kg/m^3, μ^{ex} is in units of k_BT s_{H_2} is in units of mol/L, and x_{H_2} is in units of 10^{-4}. Uncertainties in pressure and temperature are zero as these are imposed in the simulations (NPT ensemble).

T	P	m_{NaCl}	M_{NaCl}	ρ	σ_ρ	μ^{ex}	$\sigma_{\mu^{ex}}$	s_{H_2}	$\sigma_{s_{H_2}}$	x_{H_2}	$\sigma_{x_{H_2}}$
298	1	0.00	0.00	995	2	4.03	0.03	0.00072	0.00002	0.130	0.003
298	1	0.93	0.91	1032	2	4.22	0.04	0.00059	0.00002	0.107	0.004
298	1	2.96	2.79	1104	2	4.64	0.06	0.00039	0.00002	0.071	0.004
298	1	5.00	4.51	1166	2	5.04	0.03	0.00026	0.00001	0.048	0.001
298	1	5.93	5.24	1191	3	5.16	0.06	0.00023	0.00001	0.043	0.003
298	10	0.00	0.00	997	2	4.10	0.02	0.00667	0.00012	1.205	0.022
298	10	0.93	0.91	1033	2	4.26	0.03	0.00569	0.00019	1.028	0.034
298	10	2.96	2.79	1104	2	4.67	0.04	0.00377	0.00017	0.686	0.031
298	10	5.00	4.51	1166	2	5.05	0.05	0.00258	0.00012	0.472	0.022
298	10	5.93	5.24	1192	2	5.22	0.05	0.00219	0.00011	0.403	0.021
298	100	0.00	0.00	1000	2	4.13	0.02	0.06696	0.00114	12.050	0.207
298	100	0.93	0.91	1037	2	4.34	0.03	0.05401	0.00165	9.723	0.300
298	100	2.96	2.80	1108	2	4.74	0.03	0.03634	0.00099	6.576	0.184
298	100	5.00	4.52	1170	2	5.13	0.07	0.02463	0.00162	4.493	0.300
298	100	5.93	5.26	1195	2	5.25	0.06	0.02187	0.00138	4.010	0.259
298	400	0.00	0.00	1013	2	4.36	0.01	0.25620	0.00290	45.350	0.506
298	400	0.93	0.92	1049	2	4.58	0.02	0.20550	0.00350	36.450	0.626
298	400	2.96	2.82	1119	2	4.98	0.05	0.13810	0.00729	24.720	1.327
298	400	5.00	4.56	1180	2	5.39	0.04	0.09156	0.00359	16.540	0.654
298	400	5.93	5.30	1204	2	5.45	0.06	0.08646	0.00482	15.710	0.882
298	1000	0.00	0.00	1038	2	4.87	0.04	0.55910	0.02658	96.080	4.627
298	1000	0.93	0.94	1071	1	5.05	0.03	0.46960	0.01277	81.200	2.236
298	1000	2.96	2.87	1138	2	5.42	0.02	0.32260	0.00833	56.540	1.472
298	1000	5.00	4.63	1198	2	5.76	0.06	0.23010	0.01463	40.840	2.608

Table S13 continued from previous page

T	P	m_{NaCl}	M_{NaCl}	ρ	σ_ρ	μ^{ex}	$\sigma_{\mu^{\mathrm{ex}}}$	$s_{\mathrm{H_2}}$	$\sigma_{s_{\mathrm{H_2}}}$	$x_{\mathrm{H_2}}$	$\sigma_{x_{\mathrm{H_2}}}$
298	1000	5.93	5.38	1223	2	5.96	0.08	0.18870	0.01520	33.700	2.721
323	1	0.00	0.00	987	1	4.05	0.02	0.00065	0.00001	0.119	0.002
323	1	0.93	0.90	1022	1	4.23	0.04	0.00054	0.00002	0.099	0.004
323	1	2.96	2.75	1090	1	4.57	0.03	0.00039	0.00001	0.071	0.002
323	1	5.00	4.45	1150	2	4.90	0.01	0.00028	0.00000	0.051	0.001
323	1	5.93	5.17	1175	2	5.04	0.03	0.00024	0.00001	0.045	0.002
323	10	0.00	0.00	986	1	4.03	0.02	0.00662	0.00015	1.209	0.028
323	10	0.93	0.90	1022	2	4.22	0.04	0.00551	0.00024	1.006	0.045
323	10	2.96	2.75	1090	2	4.58	0.01	0.00382	0.00004	0.704	0.007
323	10	5.00	4.45	1150	2	4.92	0.02	0.00271	0.00005	0.503	0.009
323	10	5.93	5.17	1174	2	5.01	0.02	0.00248	0.00006	0.464	0.011
323	100	0.00	0.00	991	1	4.13	0.02	0.06123	0.00114	11.120	0.212
323	100	0.93	0.90	1025	1	4.29	0.01	0.05240	0.00085	9.536	0.151
323	100	2.96	2.76	1093	1	4.62	0.03	0.03753	0.00106	6.882	0.198
323	100	5.00	4.46	1153	2	4.97	0.02	0.02658	0.00060	4.920	0.112
323	100	5.93	5.18	1178	2	5.09	0.02	0.02346	0.00039	4.362	0.076
323	400	0.00	0.00	1004	2	4.36	0.02	0.23330	0.00415	41.680	0.764
323	400	0.93	0.91	1038	1	4.52	0.01	0.19820	0.00170	35.550	0.308
323	400	2.96	2.79	1105	2	4.88	0.02	0.13920	0.00324	25.210	0.599
323	400	5.00	4.50	1164	1	5.17	0.02	0.10360	0.00254	18.970	0.475
323	400	5.93	5.23	1188	1	5.27	0.03	0.09415	0.00305	17.340	0.560
323	1000	0.00	0.00	1028	1	4.80	0.02	0.53410	0.00889	92.730	1.569
323	1000	0.93	0.93	1060	1	4.97	0.02	0.45170	0.00866	78.960	1.508
323	1000	2.96	2.84	1125	2	5.30	0.01	0.32550	0.00327	57.690	0.577
323	1000	5.00	4.58	1183	1	5.60	0.05	0.23930	0.01221	43.000	2.203
323	1000	5.93	5.31	1207	2	5.71	0.03	0.21620	0.00607	39.090	1.101
343	1	0.00	0.00	976	1	3.99	0.02	0.00065	0.00001	0.120	0.002
343	1	0.93	0.89	1009	1	4.14	0.02	0.00056	0.00001	0.103	0.002
343	1	2.96	2.72	1076	1	4.46	0.02	0.00040	0.00001	0.075	0.002
343	1	5.00	4.39	1135	1	4.75	0.01	0.00030	0.00000	0.057	0.001

Table S13 continued from previous page

T	P	m_{NaCl}	M_{NaCl}	ρ	σ_ρ	μ^{ex}	$\sigma_{\mu^{\mathrm{ex}}}$	$s_{\mathrm{H_2}}$	$\sigma_{s_{\mathrm{H_2}}}$	$x_{\mathrm{H_2}}$	$\sigma_{x_{\mathrm{H_2}}}$
343	1	5.93	5.10	1160	1	4.88	0.02	0.00027	0.00001	0.050	0.001
343	10	0.00	0.00	976	1	3.98	0.02	0.00656	0.00015	1.211	0.028
343	10	0.93	0.89	1010	1	4.15	0.02	0.00553	0.00011	1.022	0.021
343	10	2.96	2.72	1077	1	4.47	0.02	0.00401	0.00009	0.747	0.016
343	10	5.00	4.39	1135	1	4.75	0.02	0.00303	0.00005	0.570	0.009
343	10	5.93	5.10	1160	2	4.90	0.03	0.00262	0.00007	0.495	0.013
343	100	0.00	0.00	980	1	4.06	0.01	0.06213	0.00077	11.410	0.142
343	100	0.93	0.89	1014	1	4.21	0.03	0.05336	0.00138	9.823	0.259
343	100	2.96	2.73	1081	1	4.53	0.02	0.03859	0.00053	7.156	0.098
343	100	5.00	4.40	1139	2	4.81	0.03	0.02918	0.00082	5.469	0.159
343	100	5.93	5.12	1163	1	4.94	0.01	0.02573	0.00037	4.846	0.071
343	400	0.00	0.00	994	1	4.29	0.02	0.23250	0.00599	41.980	1.094
343	400	0.93	0.90	1026	1	4.44	0.03	0.20190	0.00624	36.600	1.149
343	400	2.96	2.76	1092	2	4.74	0.02	0.14930	0.00347	27.360	0.645
343	400	5.00	4.45	1150	1	5.02	0.02	0.11210	0.00272	20.770	0.514
343	400	5.93	5.17	1174	1	5.14	0.03	0.09975	0.00282	18.580	0.533
343	1000	0.00	0.00	1018	1	4.74	0.01	0.52270	0.00715	91.680	1.275
343	1000	0.93	0.92	1050	1	4.88	0.02	0.45230	0.00867	79.830	1.543
343	1000	2.96	2.81	1114	1	5.15	0.03	0.34530	0.00918	61.820	1.651
343	1000	5.00	4.52	1170	1	5.44	0.02	0.26020	0.00561	47.260	1.033
343	1000	5.93	5.25	1194	1	5.57	0.01	0.22730	0.00293	41.530	0.539
363	1	0.00	0.00	962	1	3.88	0.01	0.00068	0.00001	0.128	0.002
363	1	0.93	0.87	996	2	4.04	0.02	0.00058	0.00001	0.109	0.002
363	1	2.96	2.68	1062	1	4.33	0.03	0.00044	0.00001	0.082	0.002
363	1	5.00	4.33	1120	1	4.59	0.02	0.00034	0.00001	0.064	0.001
363	1	5.93	5.03	1144	1	4.70	0.02	0.00030	0.00001	0.058	0.001
363	10	0.00	0.00	963	1	3.88	0.01	0.00681	0.00009	1.275	0.017
363	10	0.93	0.87	996	1	4.04	0.01	0.00585	0.00004	1.096	0.007
363	10	2.96	2.68	1063	1	4.34	0.02	0.00434	0.00007	0.820	0.014
363	10	5.00	4.33	1121	1	4.60	0.04	0.00333	0.00012	0.635	0.024

Table S13 continued from previous page

T	P	m_{NaCl}	M_{NaCl}	ρ	σ_ρ	μ^{ex}	$\sigma_{\mu^{\mathrm{ex}}}$	$s_{\mathrm{H_2}}$	$\sigma_{s_{\mathrm{H_2}}}$	$x_{\mathrm{H_2}}$	$\sigma_{x_{\mathrm{H_2}}}$
363	10	5.93	5.03	1144	1	4.70	0.03	0.00302	0.00008	0.579	0.016
363	100	0.00	0.00	967	1	3.95	0.01	0.06542	0.00048	12.180	0.092
363	100	0.93	0.88	1000	1	4.09	0.02	0.05661	0.00138	10.560	0.262
363	100	2.96	2.69	1066	1	4.39	0.01	0.04189	0.00053	7.879	0.103
363	100	5.00	4.35	1124	1	4.66	0.02	0.03191	0.00063	6.059	0.122
363	100	5.93	5.05	1148	1	4.78	0.01	0.02840	0.00032	5.417	0.062
363	400	0.00	0.00	981	1	4.18	0.02	0.24340	0.00504	44.500	0.945
363	400	0.93	0.89	1014	1	4.33	0.02	0.20900	0.00410	38.370	0.756
363	400	2.96	2.72	1078	2	4.59	0.04	0.16090	0.00679	29.850	1.292
363	400	5.00	4.39	1135	1	4.88	0.03	0.12140	0.00339	22.770	0.653
363	400	5.93	5.10	1159	1	4.99	0.01	0.10810	0.00129	20.400	0.247
363	1000	0.00	0.00	1006	1	4.61	0.01	0.54840	0.00619	97.300	1.110
363	1000	0.93	0.91	1037	1	4.75	0.02	0.47870	0.01033	85.470	1.862
363	1000	2.96	2.78	1101	1	5.02	0.02	0.36540	0.00707	66.160	1.266
363	1000	5.00	4.47	1157	1	5.28	0.01	0.28160	0.00435	51.720	0.805
363	1000	5.93	5.19	1180	1	5.39	0.03	0.25170	0.00623	46.520	1.172

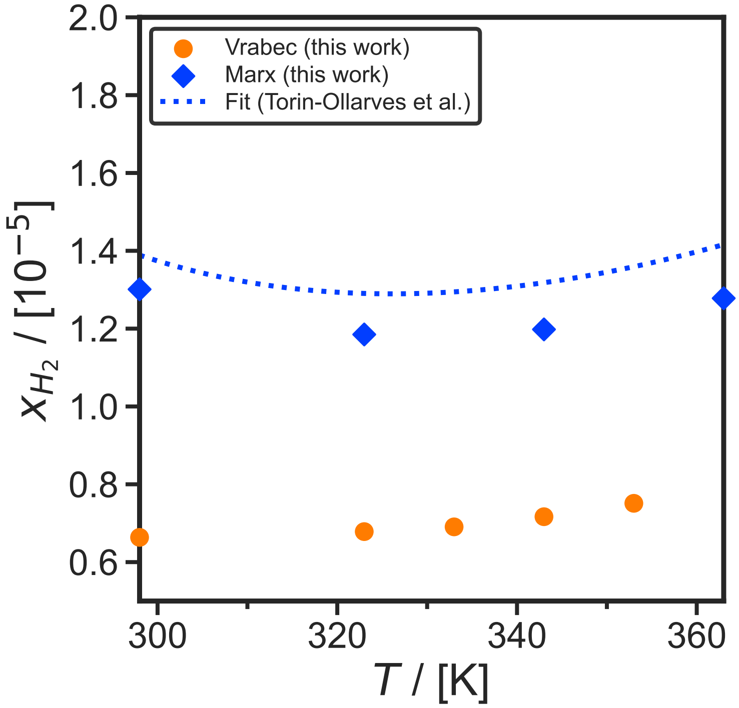

Figure S1: Computed solubilities of H_2 in pure water using the TIP4P/2005[1] H_2O force field in combination with either the Marx[2] H_2 force field (diamonds) or the Vrabec[3] force field (circles) as a function of temperature T at a H_2 partial pressure of 1 bar. The dashed lines represent the experimental correlation provided by Torín-Ollarves and Trusler[4].

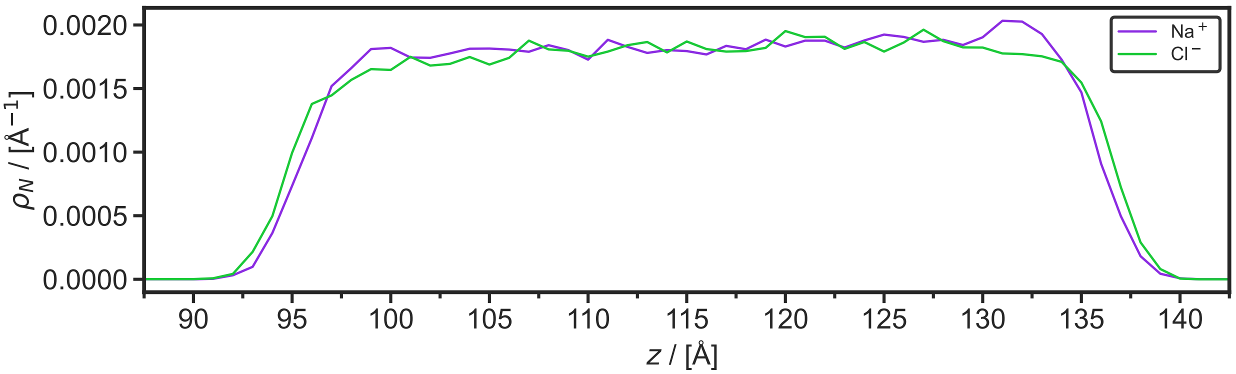

Figure S2: Number density profile ρ_N in the z-direction of Na^+ and Cl^- from a Molecular Dynamics simulation to calculate the interfacial tension of H_2 and a aqueous NaCl solution (3 mol/kg H_2O) at 343 K and 100 bar. Only the aqueous NaCl solution and the interfaces with H_2 are shown, which is part of the full simulation box. The full box is shown in Figure 1 of the main text of the manuscript. The profile is averaged for 5 ns. z is the direction perpendicular to the interface.

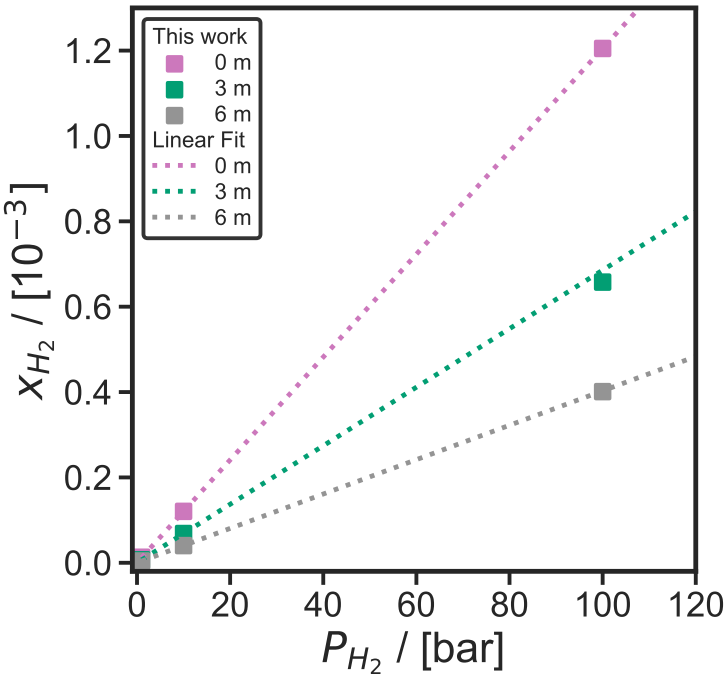

Figure S3: Computed solubilities of H_2 in aqueous NaCl solutions as a function of H_2 partial pressure at different molalities (m) of 0, 3, and 6 mol NaCl / kg H_2O. The NaCl Madrid-2019[7] force field is used in combination with the TIP4P/2005 H_2O force field[1] and the H_2 Marx force field.[2] Eq. 6-9 of the main text are used to compute the solubilities of H_2 in the aqueous solution. The solubilities are fitted by a linear function (shown as dotted lines) as described by Eq. 8 (Henry regime). Clearly, Eq. 8 is valid for H_2 partial pressures ranging from 1-100 bar.

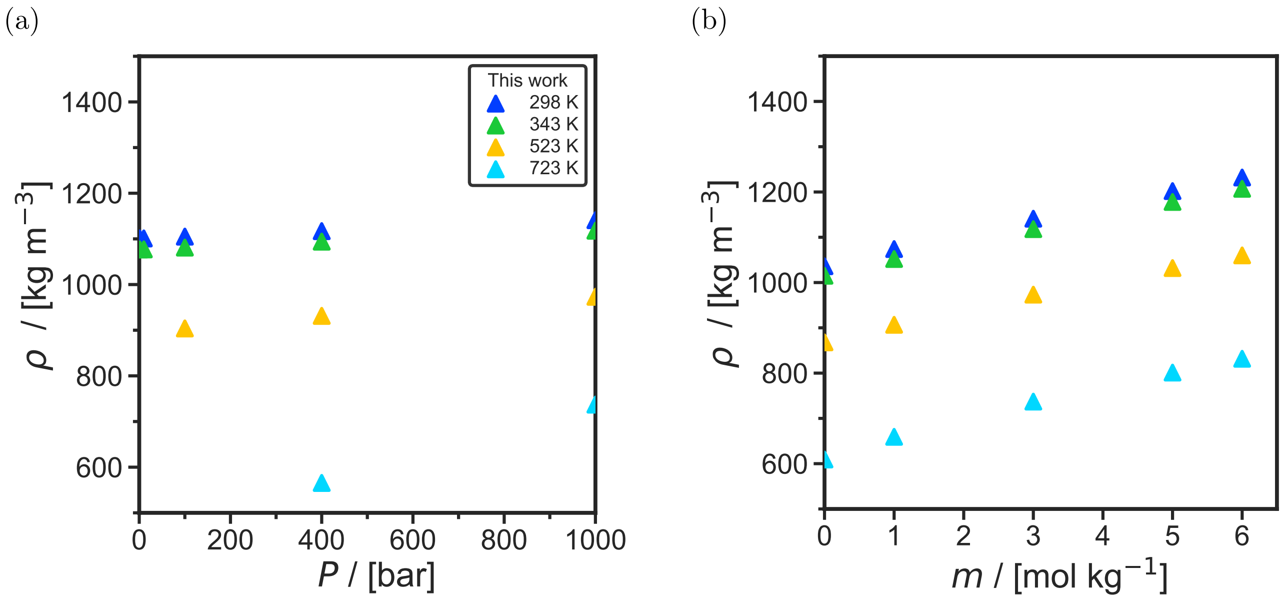

Figure S4: Densities of aqueous sodium chloride solutions as a function of (a) pressure for a solution molality of 3 mol/kg water and (b) sodium chloride molality at a pressure of 1000 bar computed using the NaCl Madrid-Transport[5,6] force field, the TIP4P/2005[1] water force field, and the Vrabec[3] hydrogen force field.

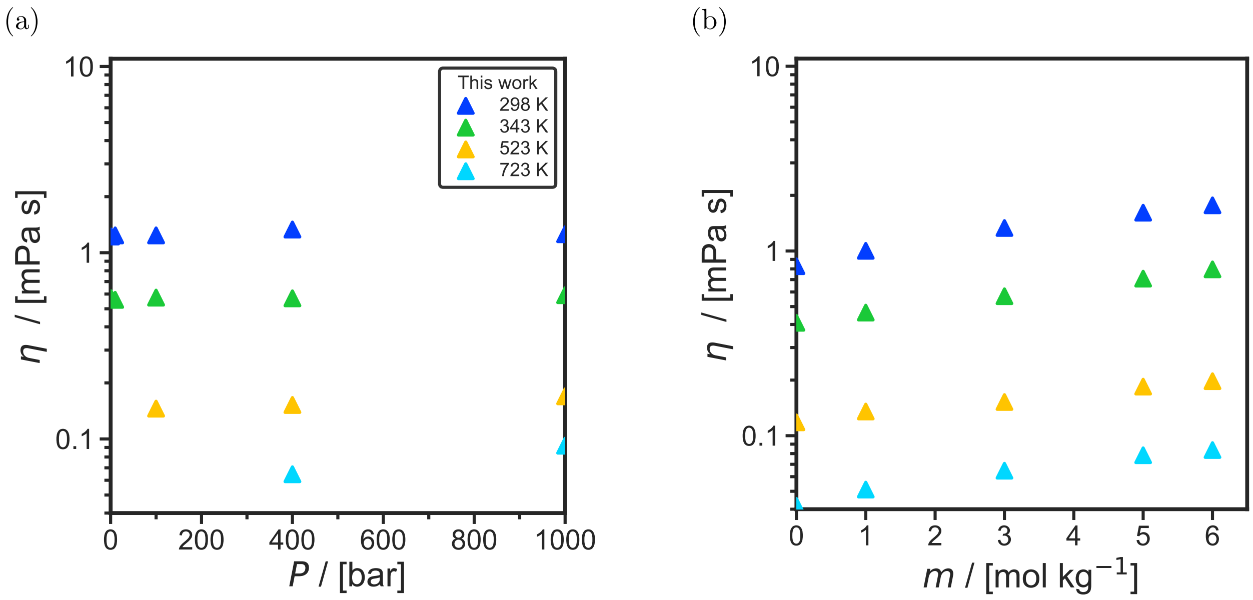

Figure S5: Viscosities of aqueous sodium chloride solutions as a function of (a) pressure at a solution molality of 3 mol NaCl/kg water and (b) sodium chloride molality computed at a pressure of 400 bar using the NaCl Madrid-Transport[5,6] force field, the TIP4P/2005[1] water force field, and the Vrabec[3] hydrogen force field.

Literature Cited

(1) Abascal, J. L.; Vega, C. A general purpose model for the condensed phases of water: TIP4P/2005. *J. Chem. Phys.* **2005**, *123*, 234505.

(2) Marx, D.; Nielaba, P. Path-integral Monte Carlo techniques for rotational motion in two dimensions: Quenched, annealed, and no-spin quantum-statistical averages. *Phys. Rev. A* **1992**, *45*, 8968.

(3) Köster, A.; Thol, M.; Vrabec, J. Molecular Models for the Hydrogen Age: Hydrogen, Nitrogen, Oxygen, Argon, and Water. *J. Chem. Eng. Data* **2018**, *63*, 305–320.

(4) Torín-Ollarves, G. A.; Trusler, J. M. Solubility of hydrogen in sodium chloride brine at high pressures. *Fluid Phase Equilib.* **2021**, *539*, 113025.

(5) Blazquez, S.; Conde, M. M.; Vega, C. Submitted. **2023**, Uploaded as Supporting Information for review only.

(6) Habibi, P.; Rahbari, A.; Blazquez, S.; Vega, C.; Dey, P.; Vlugt, T. J. H.; Moultos, O. A. A New Force Field for OH– for Computing Thermodynamic and Transport Properties of H_2 and O_2 in Aqueous NaOH and KOH Solutions. *J. Phys. Chem. B* **2022**, *126*, 9376–9387.

(7) Zeron, I. M.; Abascal, J. L. F.; Vega, C. A force field of Li^+, Na^+, K^+, Mg^{2+}, Ca^+, Cl^-, and SO_4^{2-} in aqueous solution based on the TIP4P/2005 water model and scaled charges for the ions. *J. Chem. Phys.* **2019**, *151*, 104501.

(8) Laliberté, M.; Cooper, W. E. Model for calculating the density of aqueous electrolyte solutinos. *J. Chem. Eng. Data* **2004**, *49*, 1141–1151.

(9) Laliberté, M. Model for calculating the viscosity of aqueous solutions. *J. Chem. Eng. Data* **2007**, *52*, 321–335.

(10) Celebi, A. T.; Jamali, S. H.; Bardow, A.; Vlugt, T. J. H.; Moultos, O. A. Finite-size effects of diffusion coefficients computed from molecular dynamics: a review of what we have learned so far. *Mol. Simul.* **2021**, *47*, 831–845.
